# Correction: An evolutionarily conserved role for separase in the regulation of nuclear lamins

**DOI:** 10.1038/s41420-025-02938-3

**Published:** 2026-01-28

**Authors:** Francesca Cipressa, Gaëlle Pennarun, Giuseppe Bosso, Serena Rosignoli, Liliana Tullo, Nadia Schiralli, Claudia Di Dio, Chiara Borghi, Alessandro Paiardini, Giuseppe Esposito, Michael Lewis Goldberg, Pascale Bertrand, Giovanni Cenci

**Affiliations:** 1 Department of Ecological and Biological Sciences, Università della Tuscia, Largo dell’ Università SNC, Viterbo, Italy; 2 Université Paris-Cité and Université Paris-Saclay, Inserm, CEA, UMR Stabilité Génétique Cellules Souches et Radiations, LREV/iRCM/IBFJ, Fontenay-aux-Roses, France; 3 Université Paris-Saclay, Inserm, CEA, UMR Stabilité Génétique Cellules Souches et Radiations, LREV/iRCM/IBFJ, Fontenay-aux-Roses, France; 4 Telomeres and Telomerase Group, Molecular Oncology Program, Spanish National Cancer Centre (CNIO), Madrid, Spain; 5 Centre for Regenerative Medicine “Stefano Ferrari”, Department of Life Sciences, University of Modena and Reggio Emilia, Modena, Italy; 6 Department of Biology and Biotechnologies “C. Darwin”, Sapienza Università di Roma, Rome, Italy; 7 Istituto Superiore di Sanità (ISS), Rome, Italy; 8 Stem Cell Technologies, Cambridge, UK; 9 Department of Biochemical Sciences, Sapienza University of Rome, Rome, Italy; 10 Department of Molecular Biology and Genetics, Cornell University, Ithaca, NY USA; 11 Istituto Pasteur Italia, Fondazione Cenci Bolognetti, Rome, Italy

**Keywords:** Cell division, Development

Correction to: *Cell Death Discovery* 10.1038/s41420-025-02758-5, published online 21 October 2025

During the converting process figure 4 contains blank black boxes due to the presence of layers in the file.
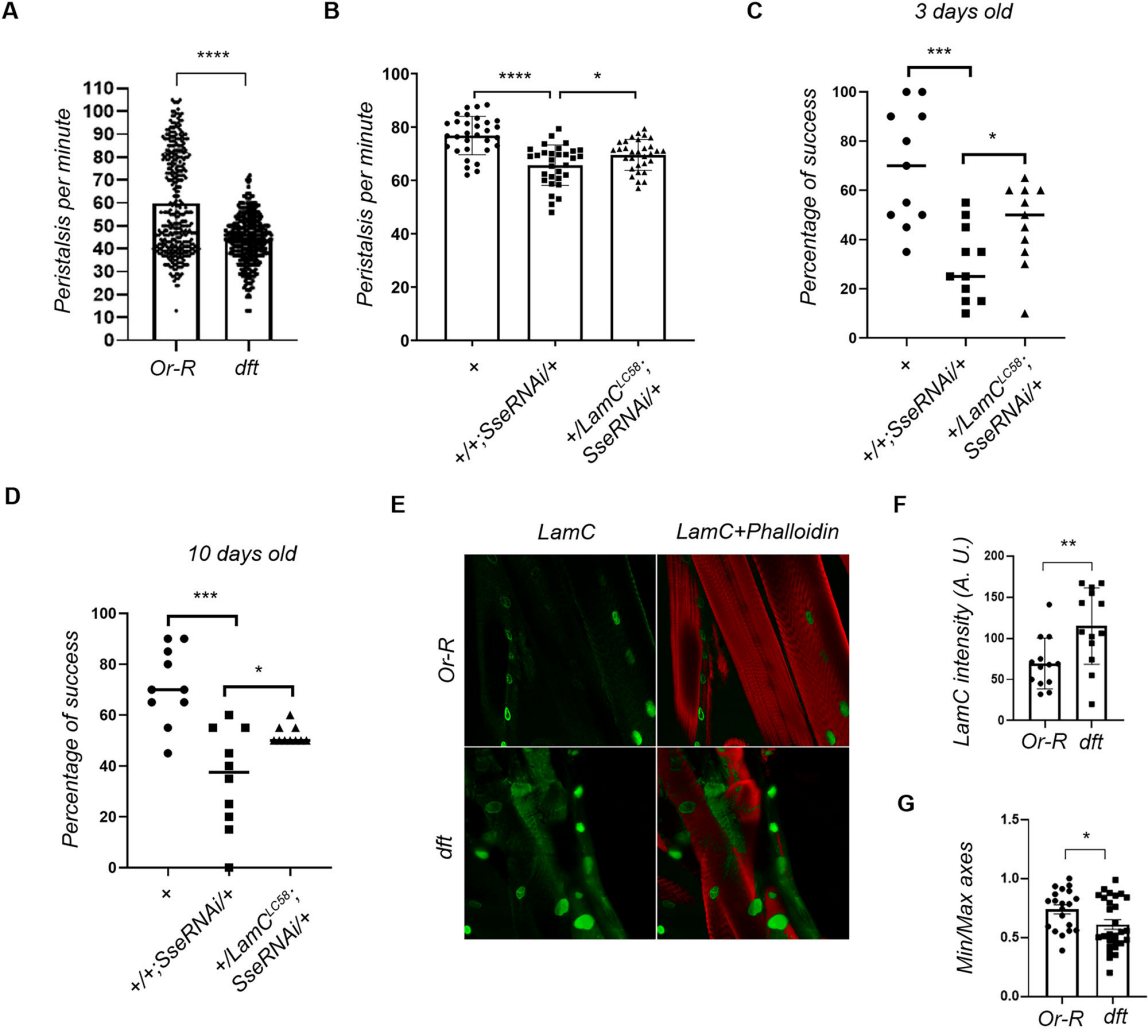


The original article has been corrected.

