# Supplementary material for: An evolutionarily conserved role for separase in the regulation of nuclear lamins
Source: Cell Death Discov. 2025 Oct 21;11:475. doi: 10.1038/s41420-025-02758-5 (PMC12540686; doi:10.1038/s41420-025-02758-5)
Supplement: Supplementary file 1 — Original Data [file 41420_2025_2758_MOESM1_ESM.docx]

**An evolutionarily conserved role for separase in the regulation of nuclear lamins**

**Original Data**

**
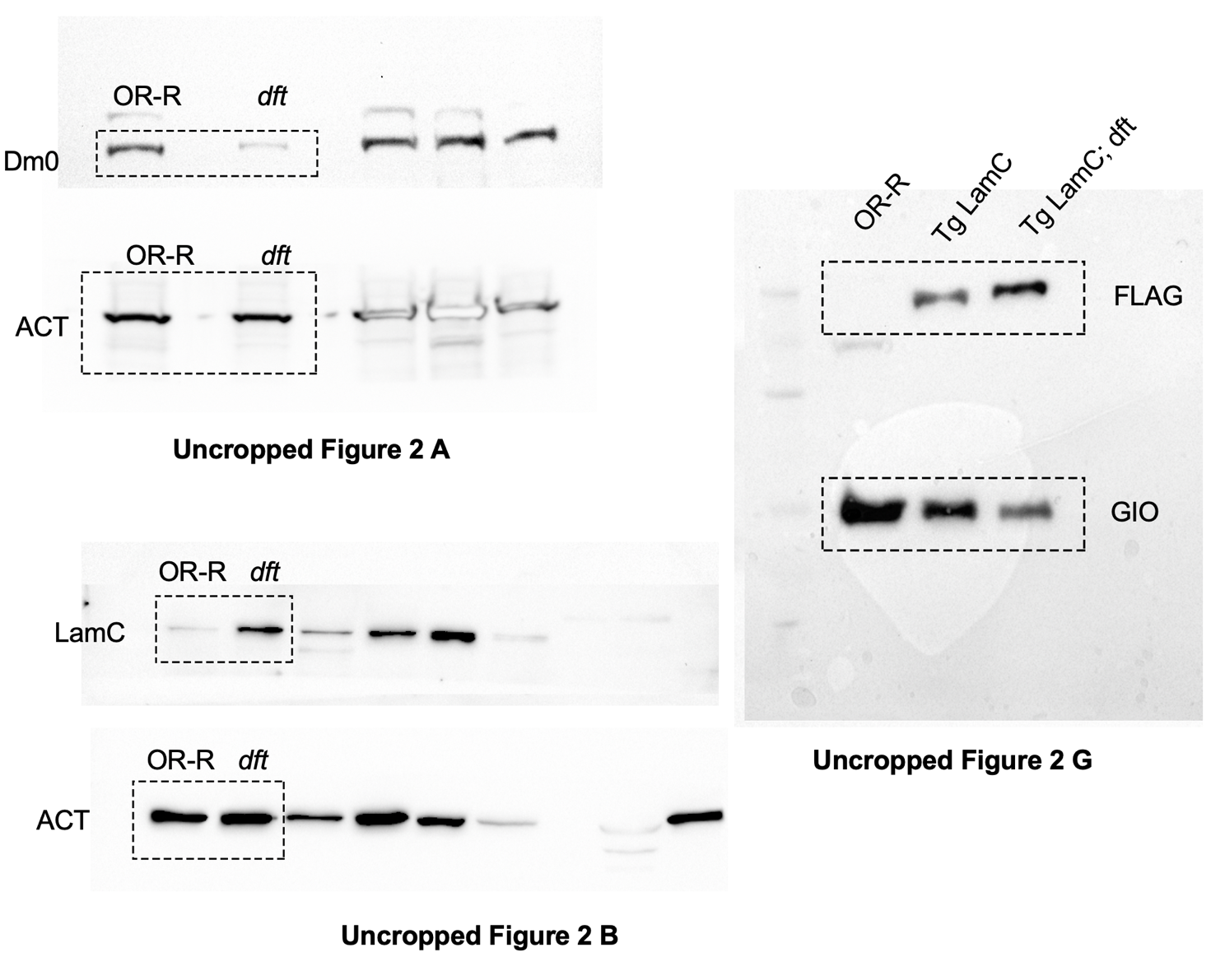
**

**Original Data Figure 1.** Uncropped blots used for Figures 2A, 2B and 2 G. Rectangles refer to the sections used for panels of Figure 3.

**
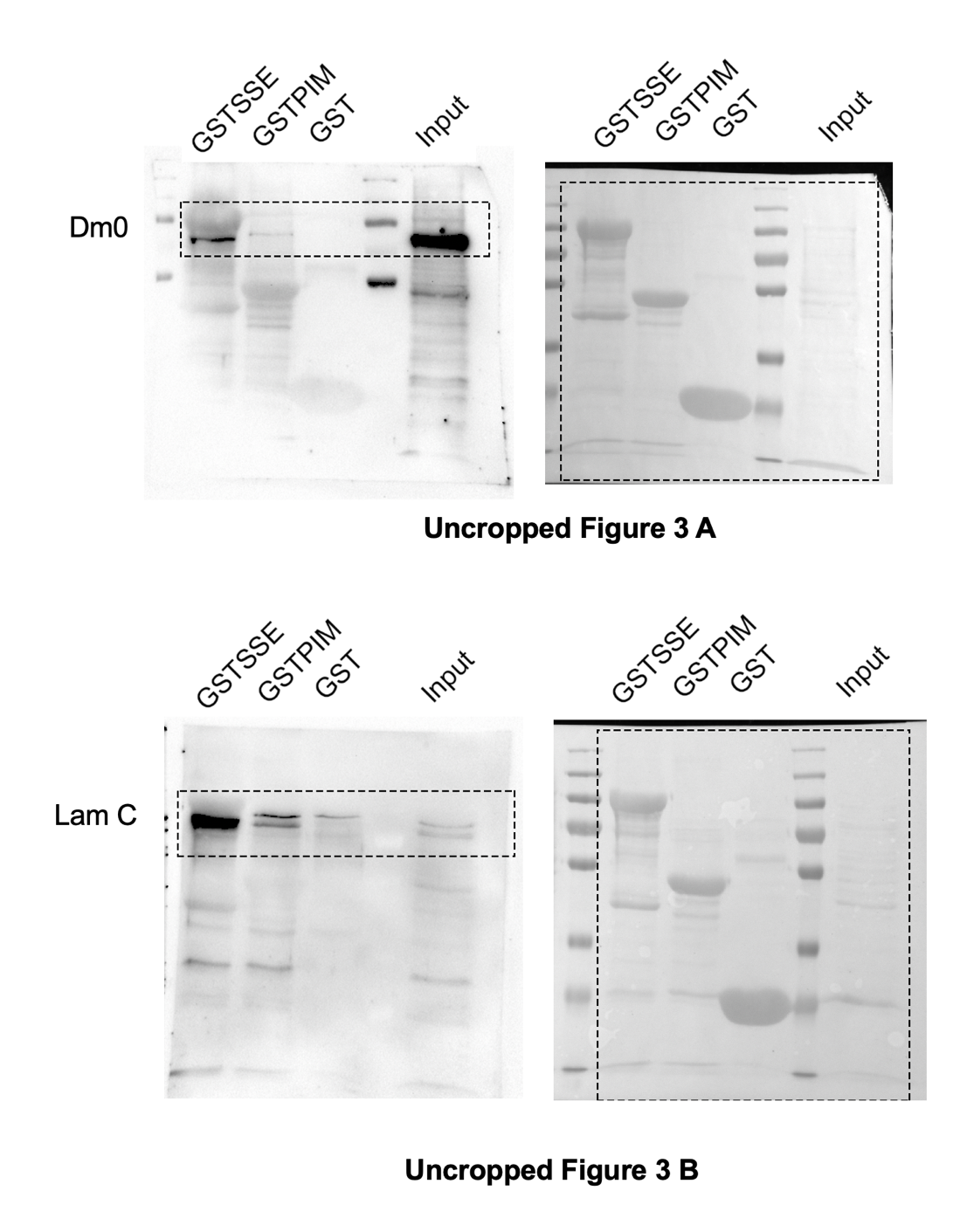
**

**Original Data Figure 2.** Uncropped blots used for Figures 3A and 3B. WBs and Ponceau are shown in the left and in the right, respectively. Rectangles refer to the sections used for panels of Figure 3.

**
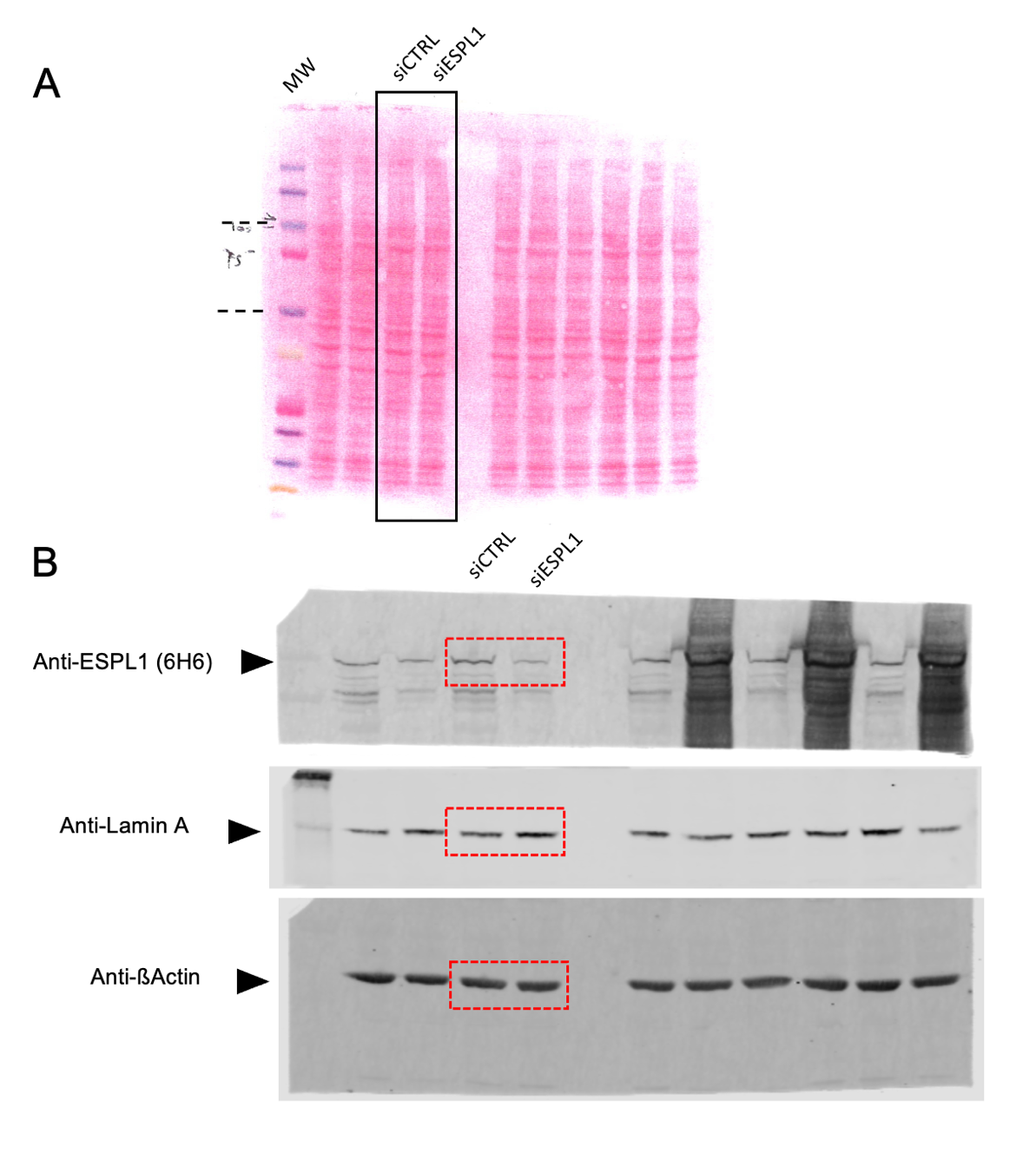
**

**Original Data Figure 3** Uncropped scan of WB shown in Figure 5B. A. Ponceau S- stained membrane immunoblotted in Figure 5B is shown. MW: Kaleidocope (Biorad). B. Scans of immunoblots shown in Figure 5B. Membrane was cutted in 3 parts according to molecular weight (> 100 kda; 50-100 kda and < 50 Kda) (dashed line indicates cut locations) and hybridized with the following antibodies, respectively: anti-ESPL1, anti-lamin A and anti-ßactin. Red rectangles referring to the sections used for panels of Figure 5B.

**
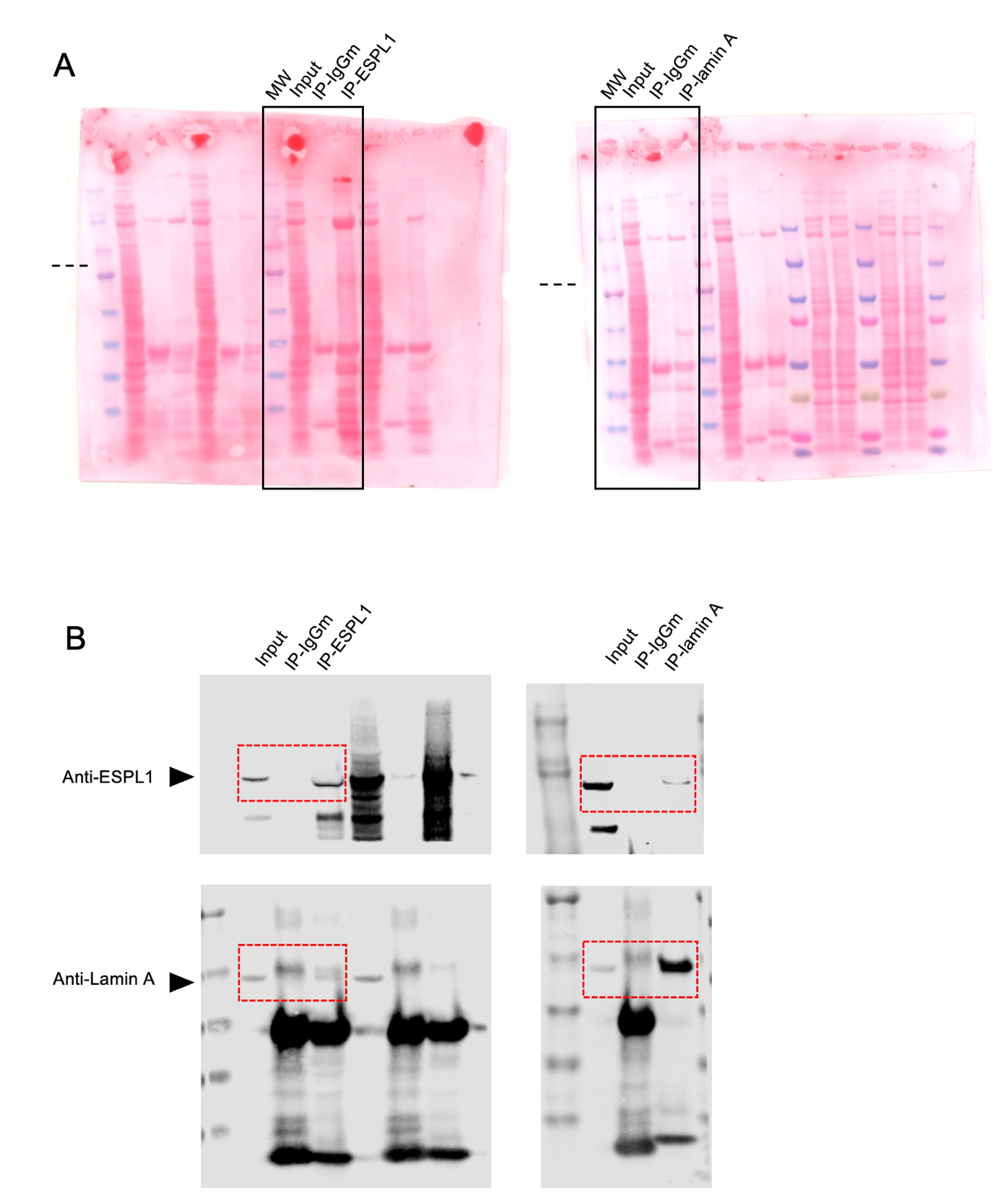
**

**Original Data Figure 4**. Uncropped scan of WB shown in Figure 6C and D. A. Ponceau S- stained membranes immunoblotted in Figure 6C (left) and D (right) are shown; MW= High Mark (Invitrogen). B. Membranes were cutted in 2 parts according to molecular weight (≥ 117 kda and ≤ 117 Kda) and hybridized with the following antibodies anti-ESPL1 and anti-lamin A ; dashed line indicates cut locations. Red rectangles refer to the sections used for panels of Figure 6C and D.
