# Supplementary material for: An evolutionarily conserved role for separase in the regulation of nuclear lamins
Source: Cell Death Discov. 2025 Oct 21;11:475. doi: 10.1038/s41420-025-02758-5 (PMC12540686; doi:10.1038/s41420-025-02758-5)
Supplement: Supplementary file 2 — Supplementary Figures [file 41420_2025_2758_MOESM2_ESM.docx]

**An evolutionarily conserved role for separase in the regulation of nuclear lamins**

**Supplementary Figures**


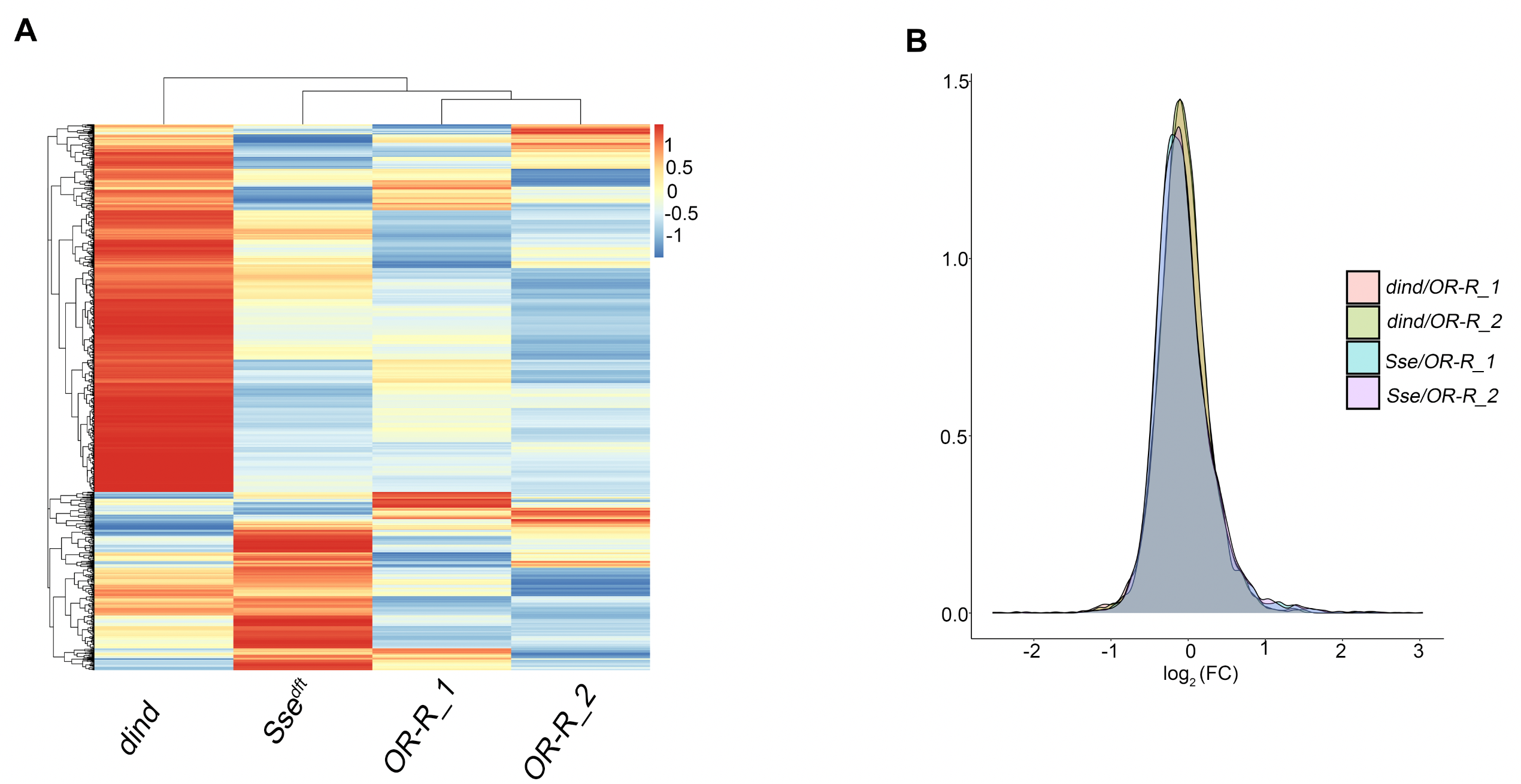


**Supplementary Figure 1**: A) Heatmap showing the log_2_ transformed abundance ratio values for the differentially expressed proteins in control (*OR-R_1* and *OR-R_2*) and in *diamond (dind)* and *Separase (Sse)* mutant extracts. B) log_2_(*FC*) values distribution. Dashed lines represent the thresholds of 0.4 and -0.4.

**
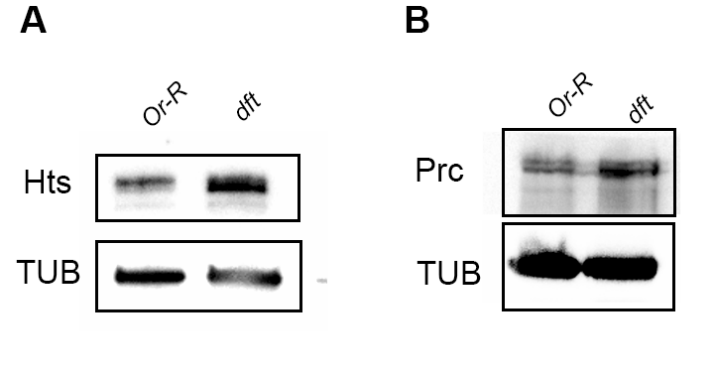
**

**Supplementary Figure 2**. WB from *Sse^dft^* mutant extracts *(dft)* showing that, consistently with our MS data (Table 1), Hts (A) and Prc (B) are upregulated upon the loss of Separase. Anti-Tubulin has been used as a loading control.


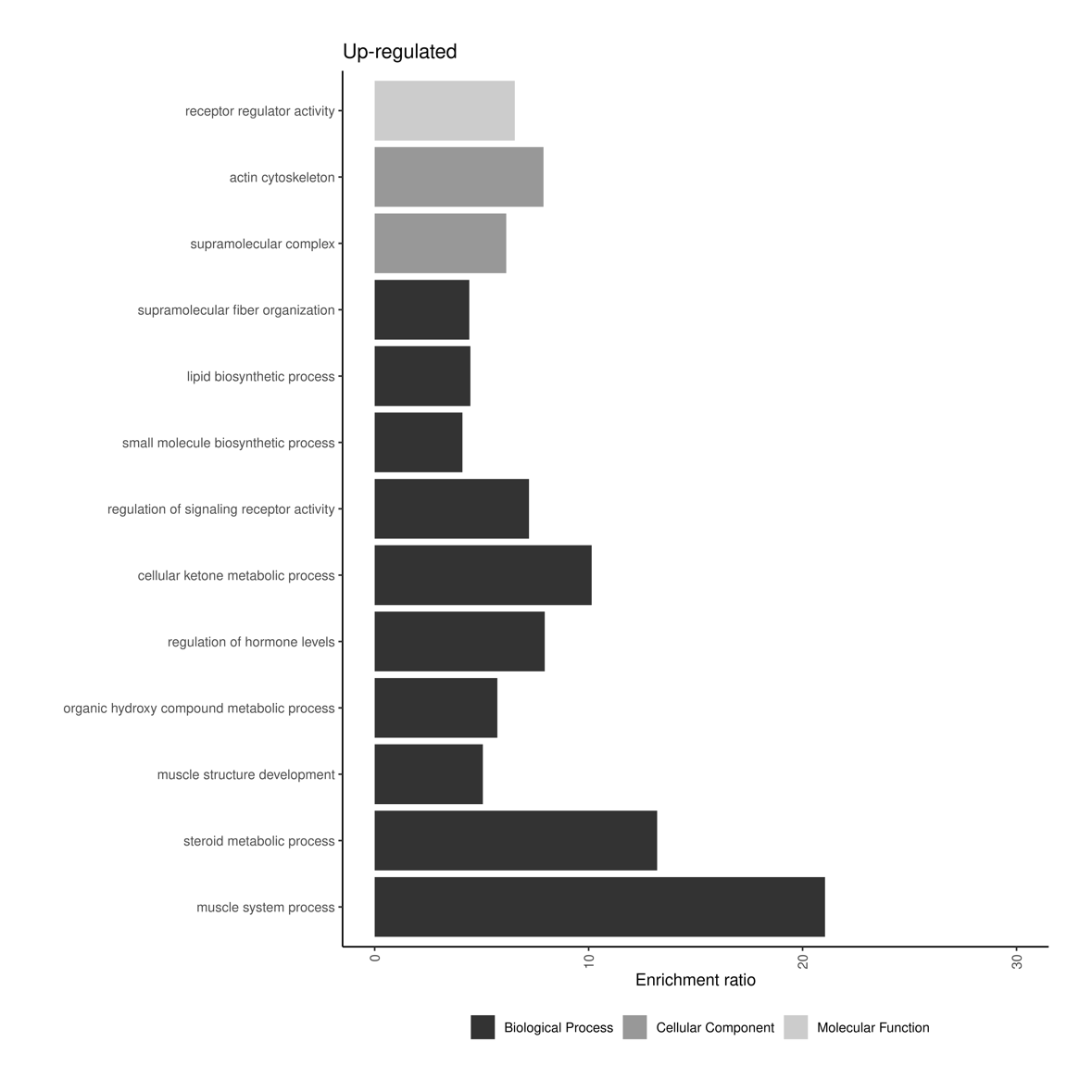


**Supplementary Figure 3**: Bar plot showing GO enriched categories of proteins up-regulated in the *Sse^dft^* mutant extracts after the elimination in the mitotic blockage mutant. All the enriched category showed an FDR < 0.05.


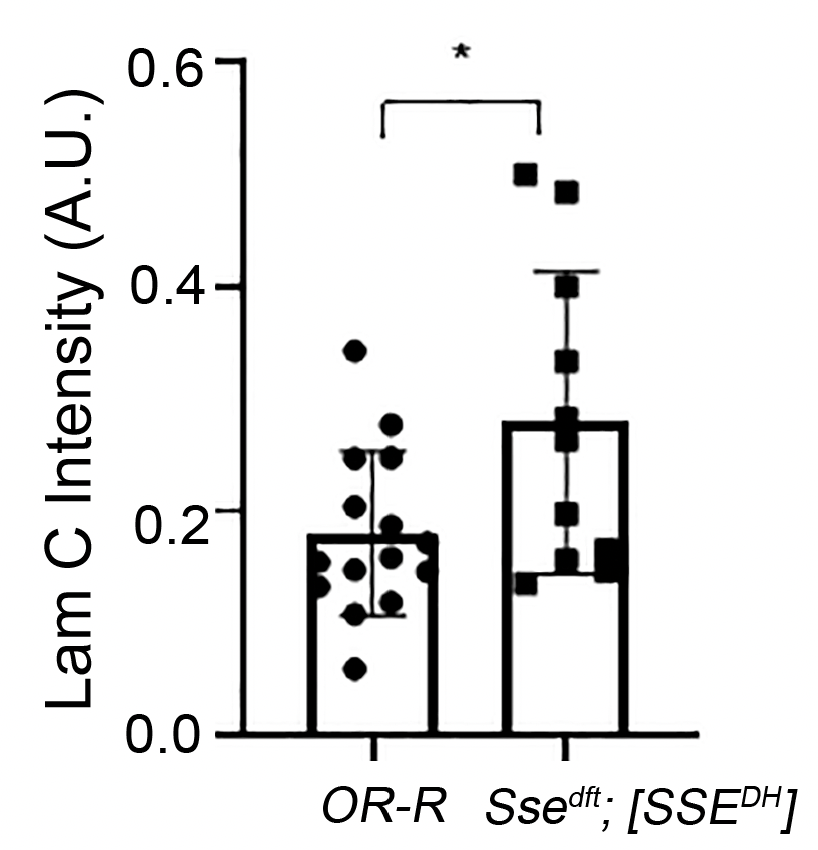


**Supplementary Figure 4**

Quantification of LamC fluorescence intensity from salivary gland immunostaining from control third instar larvae (Or-R) and *Sse^dft^* mutant larvae expressing a catalytically inactive Separase (SSE^DH^). At least three slides were used for the quantification (*p<0.05; Student’s t-test).

**
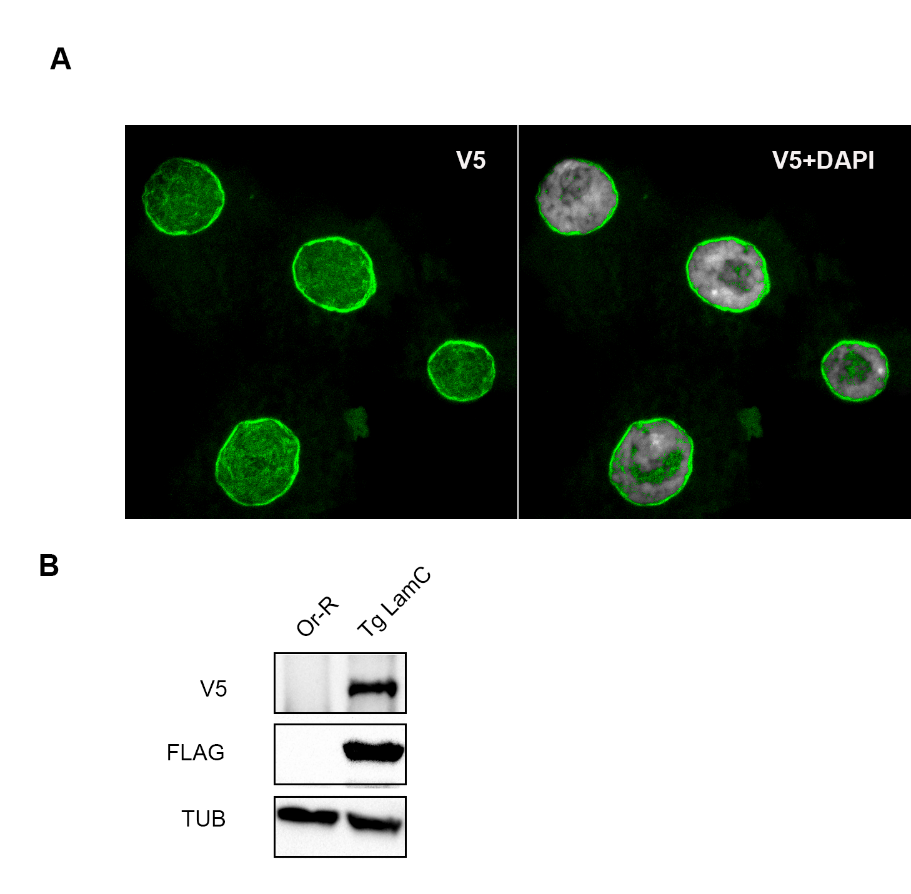
**

**Supplementary Figure 5.** Immunostaining (A) and WB (B) on larvae expressing the V5-LamC-FLAG encoding transgenes (Tg LamC) with either a commercial anti-V5 (A and B) or anti-FLAG (B) antibody. Note the perinuclear pattern of the recombinant protein in A indicating that both TAGs did not affect Lamin C expected localization.

**
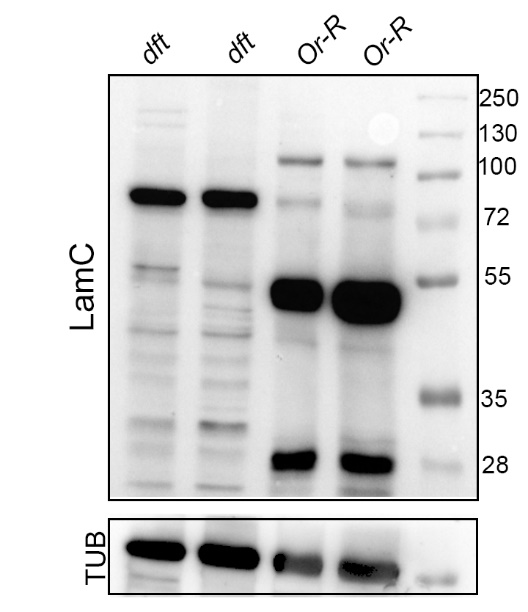
**

**Supplementary figure 6.** WB on *Sse^dft^* mutant and *OR-R* (control) larval brain extracts providing evidence of a potential SSE dependent cleavage of Lamin C that in normal conditions generates two prevalent Lam C fragments of ~50 kDa and ~25 kDa as well as a less abundant full length Lam C (~75 kDa). The cleavage is missing when Separase is lost with only the ~75 kDa full length Lamin C observable in *Sse^dft^* mutant extracts. It is worth mentioning that the presence of the two bands in control extract is consistent with the localization of one EXXR potential Separase cleavage site at position 401. Interestingly, the EXXR cleavage site at position 398-401 is also conserved in human LMNA (position 382-386) and its mutation is found in some AD-EDMD patients (R386K) ([Scharner et al., 2011](#_heading=h.gjdgxs)). We would like to mention that, for some still unclear reasons, the LamC pattern shown in this figure was not always as evident as it in all our experiments. Thus, although we are still convinced that lamin C is cleaved in physiological conditions, because of this unknown variability, we decided not to include and consider these results in our manuscript. However, we wish to show this pattern as a supplementary information hoping that this finding could provide a useful hint for all interested readers who are willing to successfully address this potential cleavage.

**
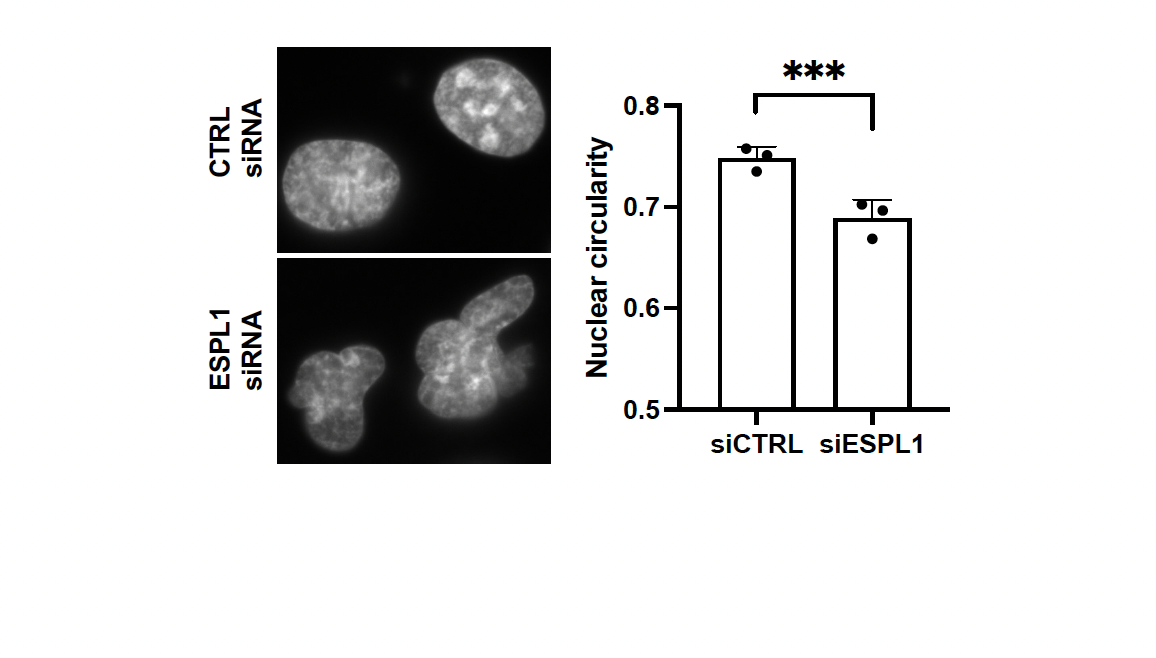
**

**Supplementary Figure 7**. DAPI staining of human fibroblast nuclei transfected with either scrambled siRNA (CTRL) or ESPL1 siRNA. Note the presence of misshapen nuclei upon ESPL1 depletion. On the right, quantification of nuclear circularity from control and ESPL1 depleted cells of 3 independent experiments (*** p<0.001).
