# Supplementary material for: An evolutionarily conserved role for separase in the regulation of nuclear lamins
Source: Cell Death Discov. 2025 Oct 21;11:475. doi: 10.1038/s41420-025-02758-5 (PMC12540686; doi:10.1038/s41420-025-02758-5)
Supplement: Supplementary file 4 — Supplementary Table 2 [file 41420_2025_2758_MOESM4_ESM.pdf]

Supplementary Table2. List of differentially expressed (down regulated) proteins in *Sse* mutants compared to *Or-R* control strain. See Text for further details

Accession	Description	Abundance.Ratio...Sse...Cntrl1	Abundance.Ratio...Sse...Cntrl2
Q59E34	CG8103-PB, isoform B OS=Drosophila melanogaster GN=Mi-2 PE=1 SV=1	-0.422752464406849	-0.473931188332412
P08928	Lamin Dm0 OS=Drosophila melanogaster GN=Lam PE=1 SV=4	-0.415037499278844	-0.426625473554056
P02572	Actin-42A OS=Drosophila melanogaster GN=Act42A PE=1 SV=3	-0.46394709975979	-0.461958546666336
D6W4T8	Coronin OS=Drosophila melanogaster GN=pod1 PE=1 SV=1	-0.508403405589552	-0.539519529959989
Q24478	Centrosome-associated zinc finger protein CP190 OS=Drosophila melanogaster GN=Cp190 PE=1 SV=2	-0.469929257774916	-0.481968507397831
M9MS15	Neurotactin, isoform C OS=Drosophila melanogaster GN=Nrt PE=4 SV=1	-117.462.139.610.707	-12.446.850.959.549
P19109	ATP-dependent RNA helicase p62 OS=Drosophila melanogaster GN=Rm62 PE=1 SV=3	-0.65744525452268	-0.63486740654747
Q9VP57	CG7752-PA OS=Drosophila melanogaster GN=pzg PE=1 SV=1	-0.403541860441014	-0.413115187147815
P09180	60S ribosomal protein L4 OS=Drosophila melanogaster GN=RpL4 PE=1 SV=2	-0.537424111957796	-0.539519529959989
A0A0B4KHI4	Syncrip, isoform Q OS=Drosophila melanogaster GN=Syp PE=1 SV=1	-152.699.243.208.383	-155.639.334.852.439
Q86BM5	A kinase anchor protein 200, isoform G OS=Drosophila melanogaster GN=Akap200 PE=1 SV=1	-0.440263475567017	-0.500217879852688
A0A0B4KFA6	CD98 heavy chain, isoform D OS=Drosophila melanogaster GN=CD98hc PE=1 SV=1	-0.422752464406849	-0.488026018218199
Q9V9U3	CG1910, isoform A OS=Drosophila melanogaster GN=CG1910 PE=1 SV=1	-0.465938397578882	-0.516635639286651
A0A0B4LEV2	Disc proliferation abnormal, isoform B OS=Drosophila melanogaster GN=dpa PE=1 SV=1	-0.623709616662948	-0.655171503002559
Q9VLL3	A kinase anchor protein 200, isoform A OS=Drosophila melanogaster GN=Akap200 PE=1 SV=3	-0.673462651860048	-0.734563103950902
Q9I7K6	Protein NASP homolog OS=Drosophila melanogaster GN=CG8223 PE=1 SV=1	-0.401634794676355	-0.483984852996335
Q24368	Chromatin-remodeling complex ATPase chain Iswi OS=Drosophila melanogaster GN=Iswi PE=1 SV=1	-0.673462651860048	-0.579921884020626
P25171	Regulator of chromosome condensation OS=Drosophila melanogaster GN=Rcc1 PE=1 SV=2	-0.454031630894707	-0.446148031818874
Q86BS3	Chromator, isoform A OS=Drosophila melanogaster GN=Chro PE=1 SV=1	-0.50635266602479	-0.588573754273535
Q9W2U4	Serine/threonine-protein phosphatase 4 regulatory subunit 2 OS=Drosophila melanogaster GN=PPP4R2r PE=1 SV=2	-0.457989644463391	-0.446148031818874
M9PDH1	Enhancer of bithorax, isoform H OS=Drosophila melanogaster GN=E(bx) PE=1 SV=1	-0.588573754273535	-0.552156355637914
M9PBD9	Ran GTPase activating protein, isoform B OS=Drosophila melanogaster GN=RanGAP PE=1 SV=1	-0.457989644463391	-0.446148031818874
P02518	Heat shock protein 27 OS=Drosophila melanogaster GN=Hsp27 PE=1 SV=2	-0.891642821909581	-0.86775220170156
Q9VL18	Probable elongation factor 1-delta OS=Drosophila melanogaster GN=eEF1delta PE=1 SV=1	-0.426625473554056	-0.473931188332412
Q9VNF6	MTA1-like, isoform A OS=Drosophila melanogaster GN=MTA1-like PE=1 SV=2	-0.440263475567017	-0.45600928033515
Q9XYU1	DNA replication licensing factor Mcm3 OS=Drosophila melanogaster GN=Mcm3 PE=1 SV=1	-0.543719518489275	-0.522840788813359
Q0E9M2	Down syndrome cell adhesion molecule 1, isoform BA OS=Drosophila melanogaster GN=Dscam1 PE=1 SV=1	-0.471928835421265	-0.446148031818874
Q9TVM2	Exportin-1 OS=Drosophila melanogaster GN=emb PE=1 SV=1	-0.432454552356253	-0.508403405589552
Q8SWU7	Obg-like ATPase 1 OS=Drosophila melanogaster GN=CG1354 PE=1 SV=1	-0.510457064357526	-0.564904848379903
P00967	Trifunctional purine biosynthetic protein adenosine-3 OS=Drosophila melanogaster GN=ade3 PE=1 SV=2	-0.531156057025363	-0.543719518489275
Q9V431	Apoptosis inhibitor 5 homolog OS=Drosophila melanogaster GN=Aac11 PE=2 SV=1	-0.442222328605074	-0.473931188332412
Q9XZ61	Ubiquitin carboxyl-terminal hydrolase OS=Drosophila melanogaster GN=Uch-L5 PE=1 SV=1	-0.415037499278844	-0.430508908041284
Q9VAA9	CG7946, isoform A OS=Drosophila melanogaster GN=CG7946 PE=1 SV=1	-0.448114896528275	-0.473931188332412
M9PJN2	Flotillin 2, isoform L OS=Drosophila melanogaster GN=Flo2 PE=1 SV=1	-0.696657605512669	-0.708396441969435
Q94920	Voltage-dependent anion-selective channel OS=Drosophila melanogaster GN=porin PE=1 SV=3	-0.504304837375931	-0.512513650651464
O61491	Flotillin-1 OS=Drosophila melanogaster GN=Flo1 PE=2 SV=1	-0.512513650651464	-0.535331732996556
Q9VMI5	CG9135 protein OS=Drosophila melanogaster GN=CG9135 PE=1 SV=1	-0.556393348524385	-0.678071905112638
P49735	DNA replication licensing factor Mcm2 OS=Drosophila melanogaster GN=Mcm2 PE=1 SV=1	-0.77349147019132	-0.736965594166206
Q9VGS2	Translationally-controlled tumor protein homolog OS=Drosophila melanogaster GN=Tctp PE=1 SV=1	-0.554273296650016	-0.556393348524385
A0A0B4LHX6	Nucleoside diphosphate kinase OS=Drosophila melanogaster GN=awd PE=1 SV=1	-0.529072742524873	-0.442222328605074
Q26365	ADP,ATP carrier protein OS=Drosophila melanogaster GN=sesB PE=2 SV=4	-0.610433188237274	-0.689659879387849
Q8MT06	Guanine nucleotide-binding protein-like 3 homolog OS=Drosophila melanogaster GN=Ns1 PE=1 SV=2	-0.446148031818874	-0.518701058452435
A0A0B4KER0	Relative of woc, isoform C OS=Drosophila melanogaster GN=row PE=1 SV=1	-0.424687669312563	-0.450084446378045
Q76NR6	Regucalcin, isoform D OS=Drosophila melanogaster GN=regucalcin PE=1 SV=1	-0.467932447710969	-0.434402824145775
Q9XZU1	Exportin-2 OS=Drosophila melanogaster GN=Cas PE=2 SV=2	-0.573466861883327	-0.582079992188035
Q9VGP4	LP08082p OS=Drosophila melanogaster GN=Ranbp9 PE=1 SV=1	-0.430508908041284	-0.428565884123491
Q7KK96	Structural maintenance of chromosomes protein OS=Drosophila melanogaster GN=SMC2 PE=1 SV=1	-0.575615328461903	-0.531156057025363
Q9VHC7	FI21236p1 OS=Drosophila melanogaster GN=rump PE=1 SV=1	-0.522840788813359	-0.508403405589552
Q9VRP2	CG10576, isoform A OS=Drosophila melanogaster GN=CG10576 PE=1 SV=1	-0.426625473554056	-0.430508908041284
O18388	Importin subunit beta OS=Drosophila melanogaster GN=Fs(2)Ket PE=2 SV=2	-0.407363571393423	-0.496142467422571
Q8SX89	Kugelkern, isoform A OS=Drosophila melanogaster GN=kuk PE=1 SV=1	-0.766111939825723	-0.732164607902385
A0A0B4KGG9	C-terminal binding protein, isoform F OS=Drosophila melanogaster GN=CtBP PE=1 SV=1	-0.416962376203336	-0.454031630894707
P48159	60S ribosomal protein L23 OS=Drosophila melanogaster GN=RpL23 PE=1 SV=2	-0.488026018218199	-0.438307278601691
Q9VWG3	40S ribosomal protein S10b OS=Drosophila melanogaster GN=RpS10b PE=1 SV=2	-0.465938397578882	-0.454031630894707
P08266	DNA-directed RNA polymerase II subunit RPB2 OS=Drosophila melanogaster GN=RpII140 PE=2 SV=2	-0.46394709975979	-0.430508908041284
Q9VGW7	Arginine methyltransferase 1 OS=Drosophila melanogaster GN=Art1 PE=1 SV=1	-0.481968507397831	-0.502259911390907
Q9VQF7	Bacchus, isoform B OS=Drosophila melanogaster GN=Bacc PE=1 SV=1	-0.483984852996335	-0.586405917590825
X2JAF4	Fasciclin 2, isoform G OS=Drosophila melanogaster GN=Fas2 PE=1 SV=1	-0.586405917590825	-0.590744853315162
Q9V491	Plexin A, isoform A OS=Drosophila melanogaster GN=PlexA PE=1 SV=1	-0.483984852996335	-0.524915117051217
Q9VYV4	Amun, isoform A OS=Drosophila melanogaster GN=Amun PE=1 SV=1	-0.45205668870965	-0.492078535042672
Q9VMR6	CG12512 OS=Drosophila melanogaster GN=CG12512-RA PE=1 SV=1	-0.520769438793664	-0.508403405589552
P11997	Larval serum protein 1 gamma chain OS=Drosophila melanogaster GN=Lsp1gamma PE=2 SV=2	-0.625934281777462	-0.715485866755755
Q9XYU0	DNA replication licensing factor Mcm7 OS=Drosophila melanogaster GN=Mcm7 PE=1 SV=1	-0.751465163861321	-0.729770092762002
P08111	Lethal(2) giant larvae protein OS=Drosophila melanogaster GN=l(2)gl PE=1 SV=2	-0.477944250839036	-0.44418384493836
A0A0C4FEI8	Granny smith, isoform F OS=Drosophila melanogaster GN=grsm PE=1 SV=1	-0.401634794676355	-0.424687669312563
Q9V461	DNA replication licensing factor Mcm6 OS=Drosophila melanogaster GN=Mcm6 PE=1 SV=1	-0.666576266274808	-0.706041020971306
Q9VGW6	DNA replication licensing factor Mcm5 OS=Drosophila melanogaster GN=Mcm5 PE=1 SV=1	-0.592919224549499	-0.541617995843987
A1Z6S7	FI02061p OS=Drosophila melanogaster GN=vimar PE=1 SV=1	-0.434402824145775	-0.477944250839036
Q9V406	Activator protein 4 OS=Drosophila melanogaster GN=crp PE=1 SV=1	-0.529072742524873	-0.573466861883327
Q9VA37	DJ-1 beta OS=Drosophila melanogaster GN=dj-1beta PE=1 SV=2	-0.448114896528275	-0.522840788813359
Q9V3I1	BcDNA.LD08534 OS=Drosophila melanogaster GN=dUTPase PE=1 SV=1	-0.42081985187285	-0.432454552356253
Q7K180	LD02709p OS=Drosophila melanogaster GN=Map60 PE=1 SV=1	-0.614845103115656	-0.625934281777462
Q59E36	REST corepressor OS=Drosophila melanogaster GN=CoRest PE=1 SV=2	-0.720231578406405	-0.533242384273829
P54397	39 kDa FK506-binding nuclear protein OS=Drosophila melanogaster GN=FK506-bp1 PE=1 SV=2	-0.535331732996556	-0.502259911390907
E1JIL4	Chromatin assembly factor 1 subunit, isoform B OS=Drosophila melanogaster GN=Caf1-55 PE=1 SV=1	-0.680382065799839	-0.678071905112638
Q9W3W8	60S ribosomal protein L17 OS=Drosophila melanogaster GN=RpL17 PE=1 SV=1	-0.403541860441014	-0.41119543298445
A4V441	Singed, isoform B OS=Drosophila melanogaster GN=sn PE=1 SV=1	-0.475936324222789	-0.554273296650016
Q8IMX4	FI04408p OS=Drosophila melanogaster GN=Rox8 PE=1 SV=1	-0.592919224549499	-0.623709616662948
Q7KSM5	Aos1 OS=Drosophila melanogaster GN=Aos1 PE=1 SV=1	-0.488026018218199	-0.469929257774916
Q7KNM2	SUMO-conjugating enzyme OS=Drosophila melanogaster GN=lwr PE=1 SV=1	-0.623709616662948	-0.694321256757713
A0A0B4KI24	Nucleoplasmin, isoform B OS=Drosophila melanogaster GN=Nlp PE=4 SV=1	-0.940644722383579	-10.086.822.430.998
Q9V3A7	Structural maintenance of chromosomes protein OS=Drosophila melanogaster GN=glu PE=1 SV=1	-0.496142467422571	-0.465938397578882
M9PI41	Sticky, isoform B OS=Drosophila melanogaster GN=sti PE=1 SV=1	-0.508403405589552	-0.500217879852688
A1Z6M0	Brahma associated protein 170kD OS=Drosophila melanogaster GN=Bap170 PE=1 SV=1	-0.558516520417355	-0.567040592723894
P84051	Histone H2A OS=Drosophila melanogaster GN=His2A PE=1 SV=2	-116.812.275.880.833	-114.241.704.461.585
Q9VSL4	Glutathione S transferase O2, isoform B OS=Drosophila melanogaster GN=GstO2 PE=1 SV=2	-0.475936324222789	-0.481968507397831
M9PFF0	60S ribosomal protein L13 OS=Drosophila melanogaster GN=RpL13 PE=1 SV=1	-0.46394709975979	-0.486004020632987
Q9VRP3	AT08565p OS=Drosophila melanogaster GN=Txl PE=1 SV=1	-0.575615328461903	-0.586405917590825
P17917	Proliferating cell nuclear antigen OS=Drosophila melanogaster GN=PCNA PE=1 SV=2	-0.500217879852688	-0.545824106814198
Q6AWJ9	Tyrosine-protein kinase-like otk OS=Drosophila melanogaster GN=otk PE=1 SV=1	-0.940644722383579	-105.889.368.905.357
Q9VXK5	Putative rRNA methyltransferase OS=Drosophila melanogaster GN=CG8939 PE=1 SV=1	-0.520769438793664	-0.597277823154395
A4UZI6	Rho1, isoform C OS=Drosophila melanogaster GN=Rho1 PE=1 SV=1	-0.510457064357526	-0.490050853695689
P05205	Heterochromatin protein 1 OS=Drosophila melanogaster GN=Su(var)205 PE=1 SV=2	-0.422752464406849	-0.442222328605074
Q24298	DE-cadherin OS=Drosophila melanogaster GN=shg PE=1 SV=2	-0.899695094204314	-0.926865295369785
O61345	Protein penguin OS=Drosophila melanogaster GN=peng PE=2 SV=1	-0.459972730742493	-0.516635639286651
Q9VH48	Probable histone-arginine methyltransferase CARMER OS=Drosophila melanogaster GN=Art4 PE=1 SV=1	-0.467932447710969	-0.467932447710969
O61305	DEAD-box helicase Dbp80 OS=Drosophila melanogaster GN=Dbp80 PE=1 SV=1	-0.577766999316952	-0.63486740654747
Q86BY9	Protein rigor mortis OS=Drosophila melanogaster GN=rig PE=1 SV=1	-0.461958546666336	-0.496142467422571
P55935	40S ribosomal protein S9 OS=Drosophila melanogaster GN=RpS9 PE=1 SV=2	-0.531156057025363	-0.610433188237274

Q9VCD8	Structural maintenance of chromosomes protein OS=Drosophila melanogaster GN=SMC1 PE=1 SV=1	-0.586405917590825	-0.477944250839036
M9NGB9	Mushroom-body expressed, isoform H OS=Drosophila melanogaster GN=mub PE=1 SV=1	-0.579921884020626	-0.535331732996556
M9PGG4	Armadillo, isoform F OS=Drosophila melanogaster GN=arm PE=4 SV=1	-0.467932447710969	-0.44418384493836
Q9V9J3	Tyrosine-protein kinase Src42A OS=Drosophila melanogaster GN=Src42A PE=2 SV=1	-0.612637459164004	-0.691988685447822
P52304	Serine/threonine-protein kinase polo OS=Drosophila melanogaster GN=polo PE=1 SV=2	-0.720231578406405	-0.768567591552035
B7Z0E2	FI18812p1 OS=Drosophila melanogaster GN=Unr PE=1 SV=1	-0.428565884123491	-0.430508908041284
Q9VCI7	LD02979p OS=Drosophila melanogaster GN=RanBP3 PE=1 SV=1	-0.465938397578882	-0.50635266602479
Q9VZE4	CG1316 OS=Drosophila melanogaster GN=CG1316 PE=1 SV=2	-0.508403405589552	-0.560642821525743
P34739	Transcription termination factor 2 OS=Drosophila melanogaster GN=lds PE=1 SV=2	-0.573466861883327	-0.687334826441606
Q9VD51	Probable ATP-dependent RNA helicase pitchoune OS=Drosophila melanogaster GN=pit PE=2 SV=2	-0.471928835421265	-0.504304837375931
Q9VXG4	Annexin B11 OS=Drosophila melanogaster GN=AnxB11 PE=2 SV=2	-0.416962376203336	-0.440263475567017
Q9W2I2	CG9752 OS=Drosophila melanogaster GN=CG9752 PE=1 SV=1	-0.539519529959989	-0.575615328461903
P16620	Tyrosine-protein phosphatase 69D OS=Drosophila melanogaster GN=Ptp69D PE=1 SV=2	-0.706041020971306	-0.67116353577046
Q9I7K0	Microtubule-associated protein Jupiter OS=Drosophila melanogaster GN=Jupiter PE=1 SV=2	-0.577766999316952	-0.552156355637914
Q9V3E9	FI17138p1 OS=Drosophila melanogaster GN=spag PE=1 SV=1	-0.403541860441014	-0.442222328605074
Q7JXU4	Nuclear GTP binding protein OS=Drosophila melanogaster GN=Ns2 PE=1 SV=1	-0.564904848379903	-0.575615328461903
P02283	Histone H2B OS=Drosophila melanogaster GN=His2B PE=1 SV=2	-13.621.579.396.759	-141.888.982.477.445
Q9VCR2	Aminoacylase-1 OS=Drosophila melanogaster GN=CG6726 PE=1 SV=2	-0.481968507397831	-0.492078535042672
E1JGK5	Mapmodulin, isoform D OS=Drosophila melanogaster GN=Mapmodulin PE=1 SV=1	-0.873027143742234	-0.883635243308215
Q9VHJ7	CG11964 OS=Drosophila melanogaster GN=CG11964 PE=1 SV=1	-0.524915117051217	-0.475936324222789
Q9W1H4	DNA ligase 1 OS=Drosophila melanogaster GN=DNA-ligI PE=1 SV=2	-0.727379545337008	-0.739372091873301
A1Z6I7	Bub1-related kinase OS=Drosophila melanogaster GN=BubR1 PE=1 SV=1	-0.477944250839036	-0.496142467422571
Q6NP69	GST-containing FLYWCH zinc-finger protein OS=Drosophila melanogaster GN=gfzf PE=1 SV=1	-0.454031630894707	-0.459972730742493
Q8INH6	CG32473, isoform C OS=Drosophila melanogaster GN=CG32473-RC PE=1 SV=1	-0.432454552356253	-0.440263475567017
Q0KIB3	CG2051, isoform A OS=Drosophila melanogaster GN=CG2051 PE=1 SV=1	-0.524915117051217	-0.547931769776189
Q9VM49	CG11266, isoform A OS=Drosophila melanogaster GN=Caper PE=1 SV=1	-0.504304837375931	-0.50635266602479
P55841	60S ribosomal protein L14 OS=Drosophila melanogaster GN=RpL14 PE=1 SV=1	-0.590744853315162	-0.446148031818874
Q94516	ATP synthase subunit b, mitochondrial OS=Drosophila melanogaster GN=ATPsynB PE=2 SV=2	-0.469929257774916	-0.44418384493836
Q9VZY0	LD45195p OS=Drosophila melanogaster GN=Non2 PE=1 SV=1	-0.617056130431009	-0.63262893435147
Q9VVI1	CG7564, isoform B OS=Drosophila melanogaster GN=CG7564 PE=1 SV=1	-0.44418384493836	-0.45600928033515
P53997	Protein SET OS=Drosophila melanogaster GN=Set PE=1 SV=2	-0.682695931638085	-0.763660460831626
Q8T8R1	CCHC-type zinc finger protein CG3800 OS=Drosophila melanogaster GN=CG3800 PE=1 SV=1	-0.430508908041284	-0.434402824145775
A0A0C4DHF6	Dystroglycan, isoform D OS=Drosophila melanogaster GN=Dg PE=1 SV=1	-0.650634722405787	-0.648371670897218
Q9VJ43	LD10783p OS=Drosophila melanogaster GN=ScpX PE=1 SV=1	-0.512513650651464	-0.569179503480228
Q7JQN4	LD15481p OS=Drosophila melanogaster GN=Rs1 PE=1 SV=1	-0.442222328605074	-0.430508908041284
Q9V9T4	ATP-dependent chromatin assembly factor large subunit OS=Drosophila melanogaster GN=Acf PE=1 SV=1	-0.689659879387849	-0.703689439291908
Q9VIP9	Condensin complex subunit 2 OS=Drosophila melanogaster GN=barr PE=1 SV=1	-0.701341684435485	-0.685013514531485
M9PCH8	Barrier to autointegration factor, isoform B OS=Drosophila melanogaster GN=baf PE=4 SV=1	-0.612637459164004	-0.601649629654035
Q9VA18	Lethal (3) 03670 OS=Drosophila melanogaster GN=l(3)03670 PE=1 SV=1	-0.496142467422571	-0.438307278601691
Q9VQI7	CG3083-PA OS=Drosophila melanogaster GN=Prx6005 PE=1 SV=1	-0.508403405589552	-0.518701058452435
P84040	Histone H4 OS=Drosophila melanogaster GN=His4 PE=1 SV=2	-0.691988685447822	-0.696657605512669
P39018	40S ribosomal protein S19a OS=Drosophila melanogaster GN=RpS19a PE=1 SV=3	-0.512513650651464	-0.496142467422571
P35875	Poly [ADP-ribose] polymerase OS=Drosophila melanogaster GN=Parp PE=2 SV=1	-0.479954975960182	-0.481968507397831
M9MRC9	Ribosomal protein L27A, isoform C OS=Drosophila melanogaster GN=RpL27A PE=3 SV=1	-0.586405917590825	-0.554273296650016
A0A0B4LFD9	Ribosomal protein S23, isoform B OS=Drosophila melanogaster GN=RpS23 PE=3 SV=1	-0.547931769776189	-0.461958546666336
Q494K2	Ctf4 OS=Drosophila melanogaster GN=Ctf4 PE=1 SV=1	-0.488026018218199	-0.46394709975979
Q9V4S8	COP9 signalosome complex subunit 7 OS=Drosophila melanogaster GN=CSN7 PE=1 SV=2	-0.486004020632987	-0.479954975960182
Q9VSI1	CG7182, isoform A OS=Drosophila melanogaster GN=CG7182 PE=1 SV=2	-0.545824106814198	-0.432454552356253
Q9VIP0	Nessun dorma, isoform A OS=Drosophila melanogaster GN=nesd PE=1 SV=1	-0.440263475567017	-0.479954975960182
Q9VEX6	AAA family protein Bor OS=Drosophila melanogaster GN=bor PE=1 SV=2	-0.486004020632987	-0.520769438793664
Q9V5M6	Longitudinals lacking protein, isoforms J/P/Q/S/Z OS=Drosophila melanogaster GN=lola PE=1 SV=4	-0.416962376203336	-0.529072742524873
Q7KQZ4	Longitudinals lacking protein, isoforms A/B/D/L OS=Drosophila melanogaster GN=lola PE=1 SV=1	-0.531156057025363	-0.573466861883327
M9MSJ1	Brain tumor, isoform E OS=Drosophila melanogaster GN=brat PE=1 SV=1	-0.780908941753803	-0.746615764199925
Q9VK58	Pih1D1, isoform D OS=Drosophila melanogaster GN=Pih1D1 PE=1 SV=5	-0.42081985187285	-0.422752464406849
Q9VX15	CG8142 OS=Drosophila melanogaster GN=CG8142 PE=1 SV=2	-0.56277226108709	-0.524915117051217
M9PET3	Simjang, isoform D OS=Drosophila melanogaster GN=simj PE=1 SV=1	-0.45205668870965	-0.446148031818874
Q9U9Q1	Replication factor C 38kD subunit, isoform A OS=Drosophila melanogaster GN=RfC38 PE=1 SV=1	-0.481968507397831	-0.504304837375931
Q9VKW3	CG5313-PA OS=Drosophila melanogaster GN=RfC3 PE=1 SV=2	-0.45600928033515	-0.550042516371997
Q7KVQ0	Probable H/ACA ribonucleoprotein complex subunit 1 OS=Drosophila melanogaster GN=CG4038 PE=2 SV=1	-0.49817873457909	-0.471928835421265
P39769	Polyhomeotic-proximal chromatin protein OS=Drosophila melanogaster GN=ph-p PE=1 SV=2	-0.678071905112638	-0.79836613883035
Q9VJ44	CG17597, isoform B OS=Drosophila melanogaster GN=CG17597 PE=1 SV=2	-0.788364746672851	-0.788364746672851
A8DYK5	CG4266, isoform B OS=Drosophila melanogaster GN=CG4266 PE=1 SV=1	-0.481968507397831	-0.436353730515936
P08985	Histone H2A.v OS=Drosophila melanogaster GN=His2Av PE=1 SV=2	-0.778432211461591	-0.741782610463982
A1Z6H7	Gp210 ortholog, isoform A OS=Drosophila melanogaster GN=Gp210 PE=1 SV=1	-0.405451450449646	-0.450084446378045
Q9VXP4	Platelet-activating factor acetylhydrolase IB subunit beta homolog OS=Drosophila melanogaster GN=Paf-AHalpha PE=1 SV=1	-0.575615328461903	-0.569179503480228
Q9VI15	CG10286 OS=Drosophila melanogaster GN=CG10286 PE=1 SV=1	-0.430508908041284	-0.416962376203336
A1Z920	FI24025p1 OS=Drosophila melanogaster GN=fra PE=1 SV=1	-0.569179503480228	-0.63262893435147
E1JJE6	CG42492, isoform C OS=Drosophila melanogaster GN=CG42492 PE=1 SV=1	-0.595096877854869	-0.795859283219775
P20353	G protein alpha i subunit OS=Drosophila melanogaster GN=Galphai PE=1 SV=2	-0.761213140412883	-0.783389931257558
Q7KJ08	Alk, isoform A OS=Drosophila melanogaster GN=Alk PE=1 SV=1	-0.486004020632987	-0.475936324222789
M9NEY8	DNA N6-methyl adenine demethylase OS=Drosophila melanogaster GN=Tet PE=1 SV=1	-0.461958546666336	-0.415037499278844
P05812	Heat shock protein 67B1 OS=Drosophila melanogaster GN=Hsp67Ba PE=3 SV=1	-0.479954975960182	-0.461958546666336
Q76NQ0	Dolichyl-diphosphooligosaccharide--protein glycosyltransferase subunit 1 OS=Drosophila melanogaster GN=CG33303 PE=1 SV=1	-0.401634794676355	-0.45205668870965
Q86B87	Modifier of mdg4 OS=Drosophila melanogaster GN=mod(mdg4) PE=1 SV=1	-0.601649629654035	-0.608232280044003
Q9W542	LD07342p OS=Drosophila melanogaster GN=mip130 PE=1 SV=3	-0.440263475567017	-0.465938397578882
M9PCL7	Sema-1a, isoform G OS=Drosophila melanogaster GN=Sema-1a PE=1 SV=1	-0.763660460831626	-0.706041020971306
Q9VL96	Pescadillo homolog OS=Drosophila melanogaster GN=CG4364 PE=1 SV=1	-0.502259911390907	-0.560642821525743
Q27601	Amidophosphoribosyltransferase OS=Drosophila melanogaster GN=Prat PE=1 SV=2	-0.520769438793664	-0.550042516371997
Q9VD52	CG6015 OS=Drosophila melanogaster GN=CG6015 PE=1 SV=1	-0.405451450449646	-0.45205668870965
Q9VQJ8	Protein-lysine N-methyltransferase CG9643 OS=Drosophila melanogaster GN=CG9643 PE=1 SV=1	-0.694321256757713	-0.63710935733414
Q9VCF8	CG5854 OS=Drosophila melanogaster GN=CG5854 PE=1 SV=1	-0.650634722405787	-0.701341684435485
P20348	Sex-regulated protein janus-A OS=Drosophila melanogaster GN=janA PE=2 SV=2	-0.662003536484984	-0.588573754273535
Q9V4D4	CG2009-PA OS=Drosophila melanogaster GN=bip2 PE=1 SV=1	-0.535331732996556	-0.490050853695689
P29617	Homeobox protein prospero OS=Drosophila melanogaster GN=pros PE=1 SV=3	-0.710755714843358	-0.678071905112638
Q9W523	Polyhomeotic distal, isoform A OS=Drosophila melanogaster GN=ph-d PE=1 SV=3	-0.708396441969435	-0.873027143742234
P23572	Cyclin-dependent kinase 1 OS=Drosophila melanogaster GN=Cdk1 PE=1 SV=1	-0.614845103115656	-0.734563103950902
Q9W260	GH03113p OS=Drosophila melanogaster GN=wrapper PE=1 SV=2	-0.741782610463982	-0.567040592723894
E1JJB2	Broad, isoform P OS=Drosophila melanogaster GN=br PE=1 SV=2	-0.808437349299244	-0.768567591552035
Q9W5W6	CG9578 OS=Drosophila melanogaster GN=CG9578 PE=1 SV=2	-0.494109070270043	-0.488026018218199
Q9VCH9	CG10375, isoform A OS=Drosophila melanogaster GN=CG10375 PE=1 SV=1	-0.595096877854869	-0.529072742524873
B7YZV6	Scm-related gene containing four mbt domains, isoform C OS=Drosophila melanogaster GN=Sfmbt PE=1 SV=1	-0.475936324222789	-0.459972730742493
Q9VE69	CG31122, isoform A OS=Drosophila melanogaster GN=CG31122 PE=1 SV=3	-0.481968507397831	-0.50635266602479
Q9VAJ2	Bub3, isoform A OS=Drosophila melanogaster GN=Bub3 PE=1 SV=1	-0.49817873457909	-0.539519529959989
Q7K7A9	Flap endonuclease 1 OS=Drosophila melanogaster GN=Fen1 PE=2 SV=1	-0.552156355637914	-0.610433188237274
Q9VDR1	Mediator of RNA polymerase II transcription subunit 25 OS=Drosophila melanogaster GN=MED25 PE=2 SV=1	-0.579921884020626	-0.533242384273829
Q9VBU4	CG11858 OS=Drosophila melanogaster GN=CG11858 PE=1 SV=1	-0.643856189774725	-0.575615328461903
Q9VND7	CG2931 OS=Drosophila melanogaster GN=CG2931 PE=1 SV=1	-0.481968507397831	-0.543719518489275
Q9V597	60S ribosomal protein L31 OS=Drosophila melanogaster GN=RpL31 PE=1 SV=1	-0.465938397578882	-0.438307278601691
Q9VUB8	CG6513-PA, isoform A OS=Drosophila melanogaster GN=endos PE=1 SV=1	-0.401634794676355	-0.42081985187285
Q7KSQ0	LD46175p OS=Drosophila melanogaster GN=sea PE=1 SV=1	-0.494109070270043	-0.450084446378045
Q9XZ14	CG9634, isoform A OS=Drosophila melanogaster GN=goe PE=1 SV=1	-0.49817873457909	-0.577766999316952

M9PBM1	U2 small nuclear riboprotein auxiliary factor 38, isoform B OS=Drosophila melanogaster GN=U2af38 PE=4 SV=1	-0.465938397578882	-0.405451450449646
Q9VUL8	LD41491p OS=Drosophila melanogaster GN=Pex3 PE=1 SV=1	-0.788364746672851	-0.744197163397282
Q9VFR0	Protein BCCIP homolog OS=Drosophila melanogaster GN=CG9286 PE=2 SV=2	-0.475936324222789	-0.422752464406849
Q7KMH5	BcDNA.LD29892 OS=Drosophila melanogaster GN=Smyd4-4 PE=1 SV=1	-0.543719518489275	-0.62148837674627
P48588	40S ribosomal protein S25 OS=Drosophila melanogaster GN=RpS25 PE=1 SV=3	-0.426625473554056	-0.520769438793664
E1JJF3	Innexin OS=Drosophila melanogaster GN=Inx2 PE=1 SV=1	-0.727379545337008	-0.849440323424619
Q9W0B6	CG17249 OS=Drosophila melanogaster GN=CG17249 PE=1 SV=1	-0.518701058452435	-0.529072742524873
Q9VHI1	Hyrax OS=Drosophila melanogaster GN=hyx PE=1 SV=1	-0.486004020632987	-0.401634794676355
Q9VLV5	Probable small nuclear ribonucleoprotein E OS=Drosophila melanogaster GN=SmE PE=1 SV=1	-0.442222328605074	-0.625934281777462
Q9VAJ1	Condensin complex subunit 1 OS=Drosophila melanogaster GN=Cap-D2 PE=1 SV=1	-0.586405917590825	-0.550042516371997
Q9V3Y4	CG6851-PA, isoform A OS=Drosophila melanogaster GN=Mtch PE=1 SV=1	-0.595096877854869	-0.42081985187285
Q8IRE4	tRNA (guanine(37)-N1)-methyltransferase OS=Drosophila melanogaster GN=CG32281 PE=2 SV=2	-0.56277226108709	-0.617056130431009
Q9V535	RNA-binding protein 8A OS=Drosophila melanogaster GN=tsu PE=1 SV=1	-0.405451450449646	-0.465938397578882
D0IQG7	Chitinase 2, isoform B OS=Drosophila melanogaster GN=Cht2 PE=1 SV=1	-0.524915117051217	-0.430508908041284
Q7K3D8	DMAP1 OS=Drosophila melanogaster GN=DMAP1 PE=1 SV=1	-0.537424111957796	-0.442222328605074
X2JCI2	Tyrosine-protein kinase OS=Drosophila melanogaster GN=Src64B PE=3 SV=1	-0.610433188237274	-0.722610301189136
O18338	CG8287-PA OS=Drosophila melanogaster GN=Rab8 PE=1 SV=1	-0.603840510926846	-0.50635266602479
Q7K2X8	Nucleoporin at 44A, isoform A OS=Drosophila melanogaster GN=Nup44A PE=1 SV=1	-0.552156355637914	-0.459972730742493
Q9VRQ2	Mad2 OS=Drosophila melanogaster GN=mad2 PE=1 SV=1	-0.628162382669579	-0.623709616662948
B7Z060	TBP-associated factor 4, isoform E OS=Drosophila melanogaster GN=Taf4 PE=1 SV=1	-0.409278229990159	-0.403541860441014
Q9VJY6	60S ribosomal protein L24 OS=Drosophila melanogaster GN=RpL24 PE=1 SV=1	-0.567040592723894	-0.442222328605074
P20193	Protein suppressor of variegation 3-7 OS=Drosophila melanogaster GN=Su(var)3-7 PE=1 SV=4	-0.450084446378045	-0.428565884123491
A0A0B4LF54	Cytochrome P450-6a17, isoform B OS=Drosophila melanogaster GN=Cyp6a17 PE=1 SV=1	-0.687334826441606	-0.641603738043346
Q7JVG2	Aps, isoform A OS=Drosophila melanogaster GN=Aps PE=1 SV=1	-0.457989644463391	-0.41888982477445
A1Z729	CG2064 OS=Drosophila melanogaster GN=CG2064 PE=1 SV=1	-0.481968507397831	-0.564904848379903
M9PEK6	Mushroom body defect, isoform L OS=Drosophila melanogaster GN=mud PE=1 SV=1	-0.703689439291908	-0.694321256757713
Q9W0G1	Tyrosine-protein phosphatase non-receptor type 61F OS=Drosophila melanogaster GN=Ptp61F PE=1 SV=1	-0.514573172829758	-0.603840510926846
Q9Y113	Negative elongation factor B OS=Drosophila melanogaster GN=NELF-B PE=1 SV=1	-0.481968507397831	-0.469929257774916
Q9VZL1	LP07226p OS=Drosophila melanogaster GN=mge PE=1 SV=1	-0.689659879387849	-0.586405917590825
Q9VMP9	Glucosamine-6-phosphate isomerase OS=Drosophila melanogaster GN=Gnpda1 PE=2 SV=1	-0.467932447710969	-0.512513650651464
Q7JVZ8	Glutathione S transferase E11, isoform A OS=Drosophila melanogaster GN=GstE11 PE=1 SV=1	-0.442222328605074	-0.407363571393423
Q9Y124	BcDNA.GH08385 OS=Drosophila melanogaster GN=BcDNA.GH08385 PE=1 SV=1	-0.428565884123491	-0.46394709975979
O62609	Mothers against decapentaplegic homolog OS=Drosophila melanogaster GN=Med PE=1 SV=1	-0.416962376203336	-0.45600928033515
Q9VJI7	CG13277-PA OS=Drosophila melanogaster GN=LSm7 PE=1 SV=1	-0.403541860441014	-0.461958546666336
A8Y560	Ribosomal protein L15 OS=Drosophila melanogaster GN=RpL15 PE=1 SV=2	-0.50635266602479	-0.475936324222789
Q9W1I6	LD12035p OS=Drosophila melanogaster GN=ytr PE=1 SV=1	-0.72499295250013	-0.698997743967186
O96692	RE63021p OS=Drosophila melanogaster GN=Rap2l PE=1 SV=1	-0.424687669312563	-0.477944250839036
P48611	6-pyruvoyl tetrahydrobiopterin synthase OS=Drosophila melanogaster GN=pr PE=1 SV=1	-0.428565884123491	-0.475936324222789
Q94513	Boundary element associated factor OS=Drosophila melanogaster GN=BEAF-32 PE=1 SV=1	-0.573466861883327	-0.438307286016091
Q9VWQ7	CG6891-PA, isoform A OS=Drosophila melanogaster GN=CG6891 PE=1 SV=1	-0.573466861883327	-0.590744853315162
Q9V3F8	Pyrroline-5-carboxylate reductase OS=Drosophila melanogaster GN=P5cr PE=1 SV=1	-0.556393348524385	-0.512513650651464
Q9VXI6	Cytochrome b-c1 complex subunit 7 OS=Drosophila melanogaster GN=UQCR-14 PE=1 SV=1	-0.426625473554056	-0.529072742524873
Q7K0L8	FLASH ortholog, isoform A OS=Drosophila melanogaster GN=FLASH PE=1 SV=1	-0.524915117051217	-0.422752464406849
O01382	Caspase OS=Drosophila melanogaster GN=Drice PE=1 SV=2	-0.50635266602479	-0.597277823154395
Q9W3D1	Caf1-180 OS=Drosophila melanogaster GN=Caf1-180 PE=1 SV=1	-0.44418384493836	-0.407363571393423
A0A0B4KHJ7	MRG15, isoform B OS=Drosophila melanogaster GN=MRG15 PE=4 SV=1	-0.490050853695689	-0.41888982477445
Q9VK57	LD15349p OS=Drosophila melanogaster GN=Pih1D1 PE=1 SV=1	-0.504304837375931	-0.529072742524873
M9PIC9	Spc105-related, isoform B OS=Drosophila melanogaster GN=Spc105R PE=1 SV=1	-0.537424111957796	-0.539519529959989
P52654	Transcription initiation factor IIA subunit 1 OS=Drosophila melanogaster GN=TfIIA-L PE=1 SV=2	-0.446148031818874	-0.41888982477445
Q9VDQ3	CG4936 OS=Drosophila melanogaster GN=CG4936 PE=1 SV=1	-0.533242384273829	-0.567040592723894
Q9W088	DNA polymerase delta small subunit OS=Drosophila melanogaster GN=CG12018 PE=2 SV=1	-0.83650126771712	-0.883635243308215
A1Z6M6	DNA polymerase interacting tpr containing protein of 47kD, isoform A OS=Drosophila melanogaster GN=Dpit47 PE=1 SV=1	-0.606034724339757	-0.573466861883327
Q9VZ62	CG11207-PA OS=Drosophila melanogaster GN=feo PE=1 SV=1	-0.595096877854869	-0.639354797539784
Q9VWA8	Protein FRG1 homolog OS=Drosophila melanogaster GN=FRG1 PE=2 SV=1	-0.42081985187285	-0.465938397578882
Q24317	DNA primase small subunit OS=Drosophila melanogaster GN=DNApol-alpha50 PE=2 SV=2	-0.915935735211525	-0.785875194647153
A1Z987	Cap-G, isoform F OS=Drosophila melanogaster GN=Cap-G PE=1 SV=2	-0.694321256757713	-0.710755714843358
Q9VBH7	CG14544 OS=Drosophila melanogaster GN=CG14544 PE=1 SV=1	-0.477944250839036	-0.588573754273535
Q9VEX5	Protein asunder OS=Drosophila melanogaster GN=Asun PE=1 SV=1	-0.547931769776189	-0.483984852996335
M9MS48	Rbp1-like, isoform B OS=Drosophila melanogaster GN=Rbp1-like PE=1 SV=1	-0.500217879852688	-0.510457064357526
Q9VHT5	LD31571p OS=Drosophila melanogaster GN=mRpL1 PE=1 SV=2	-0.473931188332412	-0.454031630894707
O02002	Caspase-1 OS=Drosophila melanogaster GN=Dcp-1 PE=1 SV=1	-0.467932447710969	-0.65744525452268
D1FYH5	Odorant-binding protein 99a OS=Drosophila melanogaster GN=Obp99a PE=4 SV=1	-0.946193556304206	-115.521.264.992.094
Q9VVL6	Mediator of RNA polymerase II transcription subunit 19 OS=Drosophila melanogaster GN=MED19 PE=2 SV=1	-0.65744525452268	-0.494109070270043
Q9VUJ0	39S ribosomal protein L39, mitochondrial OS=Drosophila melanogaster GN=mRpL39 PE=1 SV=2	-0.522840788813359	-0.512513650651464
P49846	Transcription initiation factor TFIID subunit 5 OS=Drosophila melanogaster GN=Taf5 PE=1 SV=1	-0.446148031818874	-0.446148031818874
Q9W1N3	Levy, isoform A OS=Drosophila melanogaster GN=levy PE=1 SV=1	-0.554273296650016	-0.628162382669579
Q9VGP7	Mitochondrial ribosomal protein L40 OS=Drosophila melanogaster GN=mRpL40 PE=1 SV=1	-0.554273296650016	-0.481968507397831
Q9VBP5	Jing interacting gene regulatory 1, isoform A OS=Drosophila melanogaster GN=jigr1 PE=1 SV=1	-0.625934281777462	-0.603840510926846
Q9VFB5	IP02321p OS=Drosophila melanogaster GN=Rpb7 PE=1 SV=2	-0.846843211938579	-0.891642821909581
Q9VIQ8	CG10664-PA, isoform A OS=Drosophila melanogaster GN=COX4 PE=1 SV=1	-0.678071905112638	-0.666576266274808
Q8IML6	CG11876, isoform B OS=Drosophila melanogaster GN=CG11876 PE=1 SV=1	-0.586405917590825	-0.49817873457909
Q9V3W1	CG4599-PA, isoform A OS=Drosophila melanogaster GN=Tpr2 PE=1 SV=1	-0.522840788813359	-0.459972730742493
A4V3G8	Ballchen, isoform B OS=Drosophila melanogaster GN=ball PE=1 SV=1	-0.550042516371997	-0.601649629654035
Q05783	High mobility group protein D OS=Drosophila melanogaster GN=HmgD PE=1 SV=1	-1	-10.350.469.470.992
Q9VHG5	Insulator binding factor 1, isoform A OS=Drosophila melanogaster GN=Ibf1 PE=1 SV=1	-108.008.791.132.269	-116.165.326.347.877
Q7JZD5	Dorsal interacting protein 3 OS=Drosophila melanogaster GN=Dlip3 PE=1 SV=1	-0.63262893435147	-0.556393348524385
Q9VWV8	Nitric oxide synthase-interacting protein homolog OS=Drosophila melanogaster GN=CG6179 PE=3 SV=1	-0.531156057025363	-0.675765437729469
Q9VAY7	Protein FAM50 homolog OS=Drosophila melanogaster GN=CG12259 PE=2 SV=1	-0.592919224549499	-0.603840510926846
Q9VJZ4	AT12494p OS=Drosophila melanogaster GN=ND-B22 PE=1 SV=1	-0.795859283219775	-0.763660460831626
Q9VQ35	CG17642-PA OS=Drosophila melanogaster GN=mRpL48 PE=1 SV=1	-0.601649629654035	-0.564904848379903
Q9VEL2	Brf, isoform A OS=Drosophila melanogaster GN=Brf PE=1 SV=2	-0.500217879852688	-0.575615328461903
Q9VRP5	Ubiquitin carboxyl-terminal hydrolase 36 OS=Drosophila melanogaster GN=scny PE=1 SV=3	-0.45205668870965	-0.407363571393423
Q9VX24	CG8173-PA OS=Drosophila melanogaster GN=CG8173 PE=1 SV=1	-0.673462651860048	-0.72499295250013
Q9VL16	CG5676-PA OS=Drosophila melanogaster GN=CG5676 PE=1 SV=1	-0.504304837375931	-0.436353730515936
Q9V3R3	CG3704 OS=Drosophila melanogaster GN=EG:BACR7A4.17 PE=1 SV=1	-0.432454552356253	-0.496142467422571
Q9VEX9	Histone deacetylase complex subunit SAP18 OS=Drosophila melanogaster GN=Bin1 PE=1 SV=1	-0.533242384273829	-0.539519529959989
Q9W3G1	CG10555, isoform A OS=Drosophila melanogaster GN=CG10555 PE=1 SV=1	-0.579921884020626	-0.522840788813359
Q7K2B0	Ribosomal RNA-processing protein 8 OS=Drosophila melanogaster GN=CG7137 PE=1 SV=1	-0.504304837375931	-0.500217879852688
Q9VSY1	CG4022, isoform A OS=Drosophila melanogaster GN=CG4022 PE=2 SV=1	-0.531156057025363	-0.481968507397831
M9PBC6	CG17912, isoform F OS=Drosophila melanogaster GN=BuGZ PE=1 SV=1	-0.488026018218199	-0.547931769776189
Q9VG76	C-Myc-binding protein homolog OS=Drosophila melanogaster GN=CG17202 PE=2 SV=2	-0.641603738043346	-0.614845103115656
Q9U6L4	Protein tweety OS=Drosophila melanogaster GN=tty PE=2 SV=1	-0.510457064357526	-0.547931769776189
Q9V3W2	GM23292p OS=Drosophila melanogaster GN=ND-B17 PE=1 SV=1	-0.706041020971306	-0.687334826441606
P27716	Innexin inx1 OS=Drosophila melanogaster GN=ogre PE=1 SV=1	-0.584241333477502	-0.753895990116083
P42124	Histone-lysine N-methyltransferase E(z) OS=Drosophila melanogaster GN=E(z) PE=1 SV=2	-0.469929257774916	-0.488026018218199
Q9VYY4	Cytochrome P450 4g15 OS=Drosophila melanogaster GN=Cyp4g15 PE=2 SV=1	-0.409278229990159	-0.407363571393423
M9PBW0	Ubiquitin conjugating enzyme 12, isoform B OS=Drosophila melanogaster GN=UbcE2M PE=3 SV=1	-0.816037165157405	-0.741782610463982
Q4V5H1	Peptidyl-prolyl cis-trans isomerase OS=Drosophila melanogaster GN=CG17266 PE=1 SV=1	-0.56277226108709	-0.494109070270043
Q9VDL1	UPF0483 protein CG5412 OS=Drosophila melanogaster GN=CG5412 PE=2 SV=1	-0.442222328605074	-0.56277226108709
Q9V444	Chromatin accessibility complex 14kD protein OS=Drosophila melanogaster GN=Chrac-14 PE=1 SV=1	-124.127.043.154.214	-0.910501849160897
A1Z9I6	CG13344, isoform A OS=Drosophila melanogaster GN=CG13344 PE=1 SV=1	-0.461958546666336	-0.479954975960182

Q7KRW4	CG14516, isoform B OS=Drosophila melanogaster GN=CG14516 PE=1 SV=1	-0.510457064357526	-0.617056130431009
A0A0B4KEU2	Scribbler, isoform J OS=Drosophila melanogaster GN=sbb PE=1 SV=1	-0.550042516371997	-0.619270551496451
P43332	U1 small nuclear ribonucleoprotein A OS=Drosophila melanogaster GN=snf PE=1 SV=1	-0.486004020632987	-0.543719518489275
Q9VYB1	LD33040p OS=Drosophila melanogaster GN=Ndc80 PE=1 SV=1	-0.422752464406849	-0.428565884123491
M9PBD6	Lethal (2) 37Cg, isoform D OS=Drosophila melanogaster GN=l(2)37Cg PE=1 SV=1	-0.450084446378045	-0.424687669312563
Q6NP72	CG13220, isoform A OS=Drosophila melanogaster GN=CG13220 PE=1 SV=1	-0.554273296650016	-0.558516520417355
Q24050	Anon-i1 protein OS=Drosophila melanogaster GN=anon-i1 PE=1 SV=1	-0.6943212567577713	-0.639354797539784
Q9VHM3	LD30467p OS=Drosophila melanogaster GN=M1BP PE=1 SV=1	-0.744197163397282	-0.708396441969435
Q9VGS3	RH44771p OS=Drosophila melanogaster GN=SdhC PE=1 SV=2	-0.550042516371997	-0.522840788813359
Q9VUA5	LD42058p OS=Drosophila melanogaster GN=ssp2 PE=1 SV=1	-0.436353730515936	-0.407363571393423
X2J6S0	Male-specific lethal 1, isoform B OS=Drosophila melanogaster GN=msl-1 PE=1 SV=1	-0.428565884123491	-0.535331732996556
Q9V998	Ubiquitin-like protein 5 OS=Drosophila melanogaster GN=ubl PE=3 SV=1	-0.793356776016605	-0.828793172581858
Q9VQF5	Cwc25 OS=Drosophila melanogaster GN=Cwc25 PE=1 SV=1	-0.446148031818874	-0.504304837375931
Q8IQI3	CG10984-PB, isoform B OS=Drosophila melanogaster GN=CG10984 PE=1 SV=2	-0.529072742524873	-0.675765437729469
Q7KIN0	Toll-7 OS=Drosophila melanogaster GN=Toll-7 PE=2 SV=1	-0.56277226108709	-0.564904848379903
Q9W5P1	Mediator of RNA polymerase II transcription subunit 21 OS=Drosophila melanogaster GN=MED21 PE=1 SV=1	-0.831357964441161	-0.83650126771712
Q9VXE5	Serine/threonine-protein kinase PAK mbt OS=Drosophila melanogaster GN=mbt PE=1 SV=2	-0.603840510926846	-0.628162382669579
A4V3J9	Darkener of apricot, isoform N OS=Drosophila melanogaster GN=Doa PE=1 SV=1	-0.428565884123491	-0.416962376203336
Q27272	Transcription initiation factor TFIID subunit 9 OS=Drosophila melanogaster GN=e(y)1 PE=1 SV=1	-0.454031630894707	-0.492078535042672
Q9VIJ5	LD03247p OS=Drosophila melanogaster GN=Pomp PE=1 SV=1	-0.504304837375931	-0.569179503480228
Q9VTM2	CG11652, isoform A OS=Drosophila melanogaster GN=CG11652 PE=1 SV=1	-0.426625473554056	-0.518701058452435
Q960C5	CG6860-PA, isoform A OS=Drosophila melanogaster GN=Lrch PE=1 SV=1	-0.771027430239839	-0.630393929968162
A1Z8K9	Superoxide dismutase [Cu-Zn] OS=Drosophila melanogaster GN=Sod3 PE=1 SV=1	-0.416962376203336	-0.461958546666336
Q24423	Zinc finger protein Noc OS=Drosophila melanogaster GN=noc PE=1 SV=1	-0.560642821525743	-0.701341684435485
Q8SWX6	CG11790, isoform A OS=Drosophila melanogaster GN=CG11790 PE=1 SV=1	-0.490050853695689	-0.415037499278844
Q9VUX0	CG5830, isoform A OS=Drosophila melanogaster GN=CG5830-RA PE=1 SV=2	-0.518701058452435	-0.496142467422571
Q9VMB6	CG31911-PA OS=Drosophila melanogaster GN=Ent2 PE=1 SV=1	-0.687334826441606	-0.628162382669579
Q9VLF6	UPF0585 protein CG18661 OS=Drosophila melanogaster GN=CG18661 PE=2 SV=2	-0.839079811818897	-0.736965594166206
Q9VX94	CG13001-PA OS=Drosophila melanogaster GN=CG13001 PE=1 SV=1	-0.492078535042672	-0.552156355637914
Q9VXT5	CWF19-like protein 2 homolog OS=Drosophila melanogaster GN=CG9213 PE=1 SV=2	-0.436353730515936	-0.413115187147815
Q8MKJ6	CG11777 OS=Drosophila melanogaster GN=CG11777 PE=1 SV=1	-0.6597225952233746	-0.678071905112638
P05552	Transcription factor Adf-1 OS=Drosophila melanogaster GN=Adf1 PE=2 SV=2	-0.446148031818874	-0.438307278601691
Q9VW53	CG8025-PA OS=Drosophila melanogaster GN=Mtr3 PE=1 SV=1	-0.407363571393423	-0.518701058452435
Q9V452	CG15736-PA OS=Drosophila melanogaster GN=Chrac-16 PE=1 SV=1	-0.888968687611256	-0.965784284662087
Q9W086	Mitochondrial ribosomal protein L46 OS=Drosophila melanogaster GN=mRpL46 PE=1 SV=1	-0.440263475567017	-0.520769438793664
A0A0B4K6C3	CG9603, isoform B OS=Drosophila melanogaster GN=COX7A PE=1 SV=1	-0.701341684435485	-0.780908941753803
Q8MT36	Probable histone-lysine N-methyltransferase Mes-4 OS=Drosophila melanogaster GN=Mes-4 PE=1 SV=2	-0.77349147019132	-0.935117148415146
Q9VUS0	CG7372-PA OS=Drosophila melanogaster GN=CG7372 PE=1 SV=2	-0.810966175609983	-0.805912947883698
M9PEA2	CG18292, isoform B OS=Drosophila melanogaster GN=CDK2AP1 PE=1 SV=1	-0.577766999316952	-0.582079992188035
Q9VH79	RE01104p OS=Drosophila melanogaster GN=Rpt3R PE=1 SV=2	-0.698997743967186	-0.483984852996335
Q9VKN7	Aurora kinase B OS=Drosophila melanogaster GN=ial PE=1 SV=1	-0.524915117051217	-0.502259911390907
Q9W3V9	Nuclear factor Y-box C OS=Drosophila melanogaster GN=Nf-YC PE=2 SV=1	-0.477944250839036	-0.486004020632987
Q0E8Q7	CG31731, isoform B OS=Drosophila melanogaster GN=CG31731 PE=1 SV=1	-0.422752464406849	-0.473931188332412
Q7JQG5	Caskin, isoform B OS=Drosophila melanogaster GN=ckn PE=2 SV=1	-0.409278229990159	-0.438307278601691
Q9VC49	DNA-directed RNA polymerases I, II, and III subunit RPABC5 OS=Drosophila melanogaster GN=Rpb10 PE=3 SV=1	-0.639354797539784	-0.473931188332412
Q9VPP5	Ribonuclease H2 subunit A OS=Drosophila melanogaster GN=CG13690 PE=2 SV=1	-0.646112163715093	-0.564904848379903
Q7JUZ6	DnaJ-related co-chaperone MRJ OS=Drosophila melanogaster GN=mrj PE=1 SV=1	-0.426625473554056	-0.409278229990159
Q9VG45	Dpp target protein OS=Drosophila melanogaster GN=Dtg PE=1 SV=1	-0.483984852996335	-0.826232932263294
A1ZBW7	WASH complex subunit FAM21 homolog OS=Drosophila melanogaster GN=CG16742 PE=1 SV=1	-0.512513650651464	-0.477944250839036
Q9I7M5	CG17343-PA OS=Drosophila melanogaster GN=CG17343 PE=1 SV=1	-0.630393929968162	-0.648371670897218
Q9VFJ2	39S ribosomal protein L11, mitochondrial OS=Drosophila melanogaster GN=mRpL11 PE=1 SV=1	-0.524915117051217	-0.646112163715093
Q9V877	Kinesin-like protein subito OS=Drosophila melanogaster GN=sub PE=1 SV=1	-0.531156057025363	-0.556393348524385
O76857	BCL7-like OS=Drosophila melanogaster GN=BCL7-like PE=1 SV=1	-0.46394709975979	-0.560642821525743
Q9VDB7	CG16791, isoform A OS=Drosophila melanogaster GN=CG16791 PE=1 SV=4	-0.703689439291908	-0.648371670897218
Q24297	Small nuclear ribonucleoprotein F OS=Drosophila melanogaster GN=SmF PE=1 SV=2	-0.564904848379903	-0.457989644463391
Q9W4J5	Ribosomal RNA small subunit methyltransferase NEP1 OS=Drosophila melanogaster GN=CG3527 PE=3 SV=2	-0.516635639286651	-0.514573172829758
Q9VJQ4	mRNA cap guanine-N7 methyltransferase OS=Drosophila melanogaster GN=l(2)35Bd PE=1 SV=2	-0.63710935733414	-0.573466861883327
P13008	40S ribosomal protein S26 OS=Drosophila melanogaster GN=RpS26 PE=1 SV=1	-0.486004020632987	-0.597277823154395
Q9VYW6	Rhomboid-like protein OS=Drosophila melanogaster GN=rho-4 PE=1 SV=2	-0.800877357986399	-0.778432211461591
Q9VAG5	Claret, isoform A OS=Drosophila melanogaster GN=ca PE=1 SV=4	-0.424687669312563	-0.401634794676355
Q7K332	CG30159, isoform A OS=Drosophila melanogaster GN=CG3364 PE=1 SV=1	-12.276.920.250.416	-12.584.251.525.812
E2QCK0	Transport and golgi organization 11, isoform B OS=Drosophila melanogaster GN=Tango11 PE=1 SV=1	-0.689659879387849	-0.763660460831626
Q9VJE4	DNA-directed RNA polymerase II subunit RPB11 OS=Drosophila melanogaster GN=Rpb11 PE=3 SV=1	-0.680382065799839	-0.599462070416271
Q9VUK9	CG6854-PA, isoform A OS=Drosophila melanogaster GN=CTPsyn PE=2 SV=2	-0.678071905112638	-0.558516520417355
Q9VTD6	CG43693, isoform B OS=Drosophila melanogaster GN=CG6327 PE=1 SV=1	-0.424687669312563	-0.477944250839036
Q8SWS7	LD10447p OS=Drosophila melanogaster GN=vito PE=1 SV=1	-0.526992432083826	-0.510457064357526
Q9V4C4	Synaptotagmin 7, isoform A OS=Drosophila melanogaster GN=Syt7 PE=1 SV=4	-0.666576266274808	-0.488026018218199
Q7K172	LD04933p OS=Drosophila melanogaster GN=ste24a PE=1 SV=1	-0.729770092762002	-0.756330919033137
Q9VJ62	H/ACA ribonucleoprotein complex non-core subunit NAF1 OS=Drosophila melanogaster GN=CG10341 PE=1 SV=2	-0.875671864997798	-0.675765437729469
Q5EAK6	Serine/threonine-protein kinase ATM OS=Drosophila melanogaster GN=tefu PE=2 SV=1	-0.524915117051217	-0.510457064357526
X2JHT7	Sidekick, isoform G (Fragment) OS=Drosophila melanogaster GN=sdk PE=1 SV=1	-0.488026018218199	-0.446148031818874
M9MSM5	Dishevelled associated activator of morphogenesis, isoform D OS=Drosophila melanogaster GN=DAAM PE=1 SV=1	-0.524915117051217	-0.512513650651464
Q9VPH2	DNA primase large subunit OS=Drosophila melanogaster GN=DNApol-alpha60 PE=1 SV=2	-0.45600928033515	-0.434402824145775
Q9VPB8	CG4289-PA OS=Drosophila melanogaster GN=Pex14 PE=1 SV=1	-0.434402824145775	-0.45600928033515
E1JIR3	Cdc2c, isoform C OS=Drosophila melanogaster GN=Cdk2 PE=1 SV=1	-0.880975896857725	-0.915935735211525
G2J5Z0	CG44242, isoform B OS=Drosophila melanogaster GN=CG8446-RE PE=1 SV=1	-0.584241333477502	-0.62148837674627
Q9VH69	40S ribosomal protein S29 OS=Drosophila melanogaster GN=RpS29 PE=1 SV=1	-0.675765437729469	-0.710755714843358
Q7K284	Protein CLP1 homolog OS=Drosophila melanogaster GN=cbc PE=2 SV=1	-0.494109070270043	-0.512513650651464
P13368	Protein sevenless OS=Drosophila melanogaster GN=sev PE=1 SV=2	-0.486004020632987	-0.599462070416271
Q9XZT7	Transcription initiation factor TFIID subunit 10b OS=Drosophila melanogaster GN=Taf10b PE=1 SV=1	-0.432454552356253	-0.639354797539784
Q9W0M0	CG13890 OS=Drosophila melanogaster GN=CG13890 PE=1 SV=1	-0.758769964484555	-0.788364746672851
Q95T08	CG9636, isoform C OS=Drosophila melanogaster GN=CG9636 PE=1 SV=1	-0.422752464406849	-0.573466861883327
Q9VSJ5	NADPH-dependent diflavin oxidoreductase 1 OS=Drosophila melanogaster GN=CG13667 PE=1 SV=1	-0.465938397578882	-0.508403405589552
Q9VUR7	Phosducin-like protein OS=Drosophila melanogaster GN=CG7650 PE=1 SV=2	-0.407363571393423	-0.41888982477445
Q9XYF4	Caspase Dronc OS=Drosophila melanogaster GN=Dronc PE=1 SV=1	-0.409278229990159	-0.473931188332412
Q9VAN8	CG11882, isoform A OS=Drosophila melanogaster GN=CG11882-RA PE=1 SV=1	-0.56277226108709	-0.758769964484555
Q9VRQ7	DNA polymerase epsilon subunit 2 OS=Drosophila melanogaster GN=DNApol-epsilon58 PE=1 SV=1	-0.422752464406849	-0.502259911390907
Q9VL69	CG5885-PA OS=Drosophila melanogaster GN=BEST:CK01296 PE=1 SV=1	-0.547931769776189	-0.471928835421265
Q8SYK5	Protein insensitive OS=Drosophila melanogaster GN=insv PE=1 SV=2	-0.673462651860048	-0.682695931638085
Q9VU13	CG42709, isoform A OS=Drosophila melanogaster GN=CG17667 PE=1 SV=3	-0.436353730515936	-0.49817873457909
Q8IPV3	CG3164, isoform C OS=Drosophila melanogaster GN=CG3164 PE=1 SV=1	-0.405451450449646	-0.547931769776189
Q24152	Cyclin-dependent kinases regulatory subunit OS=Drosophila melanogaster GN=Cks30A PE=3 SV=1	-0.913216233857933	-0.79836613883035
Q500Y7	CG14482, isoform A OS=Drosophila melanogaster GN=UQCR-6.4 PE=1 SV=1	-0.428565884123491	-0.42081985187285
Q9W3C8	CG11284, isoform B OS=Drosophila melanogaster GN=CG11284 PE=1 SV=1	-0.520769438793664	-0.556393348524385
Q9W3E1	GH13214p OS=Drosophila melanogaster GN=IntS4 PE=1 SV=1	-0.744197163397282	-0.603840510926846
Q9VNG0	Mediator of RNA polymerase II transcription subunit 27 OS=Drosophila melanogaster GN=MED27 PE=1 SV=1	-0.428565884123491	-0.446148031818874
P13002	Protein grainyhead OS=Drosophila melanogaster GN=grh PE=2 SV=3	-0.994240730711315	-0.921390165303633
Q9VRM6	Lethal (3) persistent salivary gland 2 OS=Drosophila melanogaster GN=l(3)psg2 PE=4 SV=2	-0.426625473554056	-0.416962376203336
B7Z043	Myocardin-related transcription factor, isoform H OS=Drosophila melanogaster GN=Mrtf PE=4 SV=3	-0.469929257774916	-0.461958546666336
X2J9B3	E2F transcription factor 2, isoform B OS=Drosophila melanogaster GN=E2f2 PE=1 SV=1	-0.430508908041284	-0.483984852996335
Q9VH38	Dimethyladenosine transferase 2, mitochondrial OS=Drosophila melanogaster GN=mtTFB2 PE=2 SV=2	-0.590744853315162	-0.586405917590825

Q9VMS1	CG14028-PA OS=Drosophila melanogaster GN=cype PE=1 SV=3	-0.63262893435147	-0.641603738043346
Q9GYU7	Mediator of RNA polymerase II transcription subunit 10 OS=Drosophila melanogaster GN=MED10 PE=1 SV=1	-0.610433188237274	-0.584241333477502
P26017	Polycomb group protein Pc OS=Drosophila melanogaster GN=Pc PE=1 SV=1	-0.608232280044003	-0.552156355637914
Q7K4H1	CG30467 OS=Drosophila melanogaster GN=CG8185 PE=1 SV=1	-0.588573754273535	-0.652901329377732
A1Z7W1	CG1868, isoform B OS=Drosophila melanogaster GN=Smyd4-1 PE=4 SV=1	-0.488026018218199	-0.516635639286651
Q9VAD6	Conserved oligomeric Golgi complex subunit 7 OS=Drosophila melanogaster GN=Cog7 PE=2 SV=2	-0.467932447710969	-0.413115187147815
E1JIB4	CG2162, isoform E OS=Drosophila melanogaster GN=CG2162 PE=4 SV=2	-0.57132159005177	-0.440263475567017
A1Z898	Caf1-105 OS=Drosophila melanogaster GN=Caf1-105 PE=1 SV=1	-0.666576266274808	-0.63262893435147
Q9VY72	Maternal gene required for meiosis, isoform H OS=Drosophila melanogaster GN=mamo PE=1 SV=4	-0.98564470702293	-0.994240730711315
P00408	Cytochrome c oxidase subunit 2 OS=Drosophila melanogaster GN=mt:CoII PE=3 SV=1	-108.314.123.530.025	-109.541.956.507.868
Q7KBL8	Mediator of RNA polymerase II transcription subunit 29 OS=Drosophila melanogaster GN=ix PE=1 SV=1	-0.465938397578882	-0.471928835421265
E1JIP3	WRN exonuclease, isoform B OS=Drosophila melanogaster GN=WRNexo PE=1 SV=1	-0.932361283124637	-0.746615764199925
Q9W2I0	CG42672, isoform P OS=Drosophila melanogaster GN=CG42672 PE=1 SV=5	-0.401634794676355	-0.403541860441014
Q9W0P3	Mediator of RNA polymerase II transcription subunit 30 OS=Drosophila melanogaster GN=MED30 PE=1 SV=1	-0.457989644463391	-0.473931188332412
P49906	Transcription initiation factor TFIID subunit 11 OS=Drosophila melanogaster GN=Taf11 PE=1 SV=1	-130.044.836.747.691	-12.584.251.525.812
Q8T9H8	CG31908, isoform B OS=Drosophila melanogaster GN=ade3 PE=2 SV=1	-109.850.554.495.243	-108.008.791.132.269
Q9VD55	Cytochrome c heme lyase, isoform A OS=Drosophila melanogaster GN=Cchl PE=1 SV=1	-0.753895990116083	-0.623709616662948
Q9W554	CG14814, isoform B OS=Drosophila melanogaster GN=CG14814 PE=1 SV=1	-148.196.850.739.783	-136.959.452.851.768
P56175	Probable RNA 3'-terminal phosphate cyclase-like protein OS=Drosophila melanogaster GN=Rtc1 PE=2 SV=3	-0.41119543298445	-0.428565884123491
Q9W0S5	Miple, isoform A OS=Drosophila melanogaster GN=miple1 PE=2 SV=2	-0.682695931638085	-0.713118852211838
Q6IKC0	CG42394, isoform A OS=Drosophila melanogaster GN=CG42394 PE=1 SV=1	-0.440263475567017	-0.403541860441014
Q9W020	Nucleolar MIF4G domain-containing protein 1 homolog OS=Drosophila melanogaster GN=CG9004 PE=2 SV=1	-0.446148031818874	-0.543719518489275
Q6IDF5	CG12859 OS=Drosophila melanogaster GN=ND-B15 PE=1 SV=1	-0.488026018218199	-0.533242384273829
Q9W141	Putative ATP synthase subunit f, mitochondrial OS=Drosophila melanogaster GN=CG4692 PE=1 SV=1	-0.746615764199925	-0.696657605512669
Q9W547	LD21404p OS=Drosophila melanogaster GN=mRpL16 PE=1 SV=1	-0.518701058452435	-0.486004020632987
Q9VM17	SOSS complex subunit B homolog OS=Drosophila melanogaster GN=CG5181 PE=2 SV=1	-0.535331732996556	-0.608232280044003
A0A0B4KFV4	CG15107, isoform B OS=Drosophila melanogaster GN=CG15107 PE=1 SV=1	-0.810966175609983	-0.778432211461591
Q9VQY9	Probable DNA replication complex GINS protein PSF2 OS=Drosophila melanogaster GN=Psf2 PE=2 SV=1	-0.438307278601691	-0.440263475567017
Q9W282	tRNA pseudouridine synthase OS=Drosophila melanogaster GN=CG3045 PE=1 SV=1	-1,98850436116217	-2,07704103576383
Q9V4B6	CG31998-PA OS=Drosophila melanogaster GN=CG31998 PE=1 SV=2	-0.558516520417355	-0.520769438793664
Q9W3S4	CG4617, isoform A OS=Drosophila melanogaster GN=CG4617 PE=1 SV=2	-0.454031630894707	-0.514573172829758
A1ZAW5	Methylosome subunit pICln OS=Drosophila melanogaster GN=icln PE=1 SV=1	-0.543719518489275	-0.481968507397831
Q9VNP3	Mesoderm-expressed 2, isoform B OS=Drosophila melanogaster GN=Mes2 PE=1 SV=1	-0.703689439291908	-0.727379545337008
Q8SZR6	CG31223 OS=Drosophila melanogaster GN=syndapin PE=1 SV=1	-0.461958546666336	-0.56277226108709
Q7JZ53	CG4866 OS=Drosophila melanogaster GN=CG4866 PE=1 SV=1	-0.652901329377732	-0.706041020971306
Q9VAS7	Innexin inx3 OS=Drosophila melanogaster GN=Inx3 PE=1 SV=1	-0.935117148415146	-0.862496476250065
Q9VMJ5	Beta galactosidase, isoform A OS=Drosophila melanogaster GN=Gal PE=1 SV=2	-0.461958546666336	-0.500217879852688
Q9VVX0	Protein Gemin2 OS=Drosophila melanogaster GN=Gem2 PE=1 SV=1	-0.407363571393423	-0.502259911390907
Q07886	Probable ATP-dependent RNA helicase Dbp45A OS=Drosophila melanogaster GN=Dbp45A PE=2 SV=2	-0.63710935733414	-0.560642821525743
Q9VM45	Nuf2 OS=Drosophila melanogaster GN=Nuf2 PE=4 SV=2	-0.965784284662087	-0.951763814347152
Q9VCG0	CG13599 OS=Drosophila melanogaster GN=CG13599 PE=1 SV=1	-0.599462070416271	-0.558516520417355
Q9VMH4	CG9175, isoform A OS=Drosophila melanogaster GN=CG9175-RA PE=1 SV=1	-0.46394709975979	-0.446148031818874
Q9VIK0	CG9319, isoform B OS=Drosophila melanogaster GN=CG9319-RA PE=1 SV=1	-0.590744853315162	-0.524915117051217
A1ZAK1	FI09622p OS=Drosophila melanogaster GN=IntS8 PE=1 SV=1	-0.524915117051217	-0.664288089679538
Q9VLN0	CG13390 OS=Drosophila melanogaster GN=CG13390 PE=4 SV=1	-0.438307278601691	-0.601649629654035
Q9NK54	Protein chiffon OS=Drosophila melanogaster GN=chif PE=1 SV=2	-0.67116353577046	-0.617056130431009
O46079	CG3587-PA OS=Drosophila melanogaster GN=EG:39E1.2 PE=1 SV=1	-220.423.305.221.761	-212.029.423.371.771
Q8IPS4	CG11109, isoform B OS=Drosophila melanogaster GN=CG11109-RB PE=1 SV=1	-0.531156057025363	-0.494109070270043
Q8IMI5	FI04474p OS=Drosophila melanogaster GN=spdo PE=2 SV=1	-0.560642821525743	-0.436353730515936
Q2PDX2	CG34001, isoform B OS=Drosophila melanogaster GN=CG34001 PE=1 SV=2	-0.678071905112638	-0.582079992188035
O17468	Protein HIRA homolog OS=Drosophila melanogaster GN=Hira PE=1 SV=2	-0.968604803724466	-0.749038426466781
A0A0B4KEN4	Van gogh, isoform B OS=Drosophila melanogaster GN=Vang PE=4 SV=1	-0.448114896528275	-0.592919224549499
Q9VTY6	Ubiquitin-conjugating enzyme E2 C OS=Drosophila melanogaster GN=vih PE=1 SV=1	-0.477944250839036	-0.508403405589552
Q9VH45	CG5359, isoform A OS=Drosophila melanogaster GN=Dlc90F PE=1 SV=1	-0.680382065799839	-0.816037165157405
M9PFW3	Frizzled 2, isoform F OS=Drosophila melanogaster GN=fz2 PE=4 SV=1	-0.63710935733414	-0.586405917590825
Q8IP99	Transcriptional adaptor 1-1, isoform A OS=Drosophila melanogaster GN=Ada1-1 PE=4 SV=1	-0.558516520417355	-0.430508908041284
Q9W4L1	39S ribosomal protein L33, mitochondrial OS=Drosophila melanogaster GN=mRpL33 PE=3 SV=2	-0.432454552356253	-0.422752464406849
Q9VSW5	Kinesin-like protein OS=Drosophila melanogaster GN=Klp67A PE=1 SV=1	-0.415037499278844	-0.45205668870965
M9NEL5	Erect wing, isoform K OS=Drosophila melanogaster GN=ewg PE=4 SV=1	-0.496142467422571	-0.434402824145775
Q7JW61	CG8494 OS=Drosophila melanogaster GN=Usp20-33 PE=1 SV=1	-0.514573172829758	-0.45205668870965
E1JIZ7	CG42487 OS=Drosophila melanogaster GN=CG42487 PE=1 SV=1	-0.768567591552035	-0.826232932263294
Q9VG07	CG7488 OS=Drosophila melanogaster GN=CG7488 PE=1 SV=2	-0.65744525452268	-0.751465163861321
A0A0B4JD21	CG10253, isoform B OS=Drosophila melanogaster GN=CG10253 PE=1 SV=1	-0.768567591552035	-0.803392955905182
Q7JWH6	CG1888, isoform A OS=Drosophila melanogaster GN=CG1888 PE=1 SV=1	-0.753895990116083	-0.639354797539784
Q9VAQ5	Probable dimethyladenosine transferase OS=Drosophila melanogaster GN=CG11837 PE=2 SV=1	-0.552156355637914	-0.518701058452435
Q9VHW6	CG10903, isoform A OS=Drosophila melanogaster GN=CG10903 PE=1 SV=1	-0.522840788813359	-0.541617995843987
M9PIU6	Zeste, isoform C OS=Drosophila melanogaster GN=z PE=4 SV=1	-114.880.066.140.671	-105.889.368.905.357
Q0KI97	CG34117 OS=Drosophila melanogaster GN=CG34117 PE=1 SV=1	-0.582079992188035	-0.575615328461903
Q9VRH6	Translation factor waclaw, mitochondrial OS=Drosophila melanogaster GN=waw PE=3 SV=2	-0.564904848379903	-0.541617995843987
C8VV95	Daughterless, isoform B OS=Drosophila melanogaster GN=da PE=1 SV=1	-0.516635639286651	-0.617056130431009
Q9XZ53	Oligosaccharyl transferase 3 OS=Drosophila melanogaster GN=OstStt3 PE=1 SV=1	-0.763660460831626	-0.739372091873301
Q9VL63	UPF0430 protein CG31712 OS=Drosophila melanogaster GN=CG31712 PE=1 SV=3	-0.520769438793664	-0.403541860441014
Q9W256	IP14609p OS=Drosophila melanogaster GN=Mes4 PE=1 SV=1	-0.554273296650016	-0.502259911390907
Q9VKC2	Amino acid transporter protein JHI-21 OS=Drosophila melanogaster GN=JhI-21 PE=1 SV=1	-0.524915117051217	-0.510457064357526
Q9V6K3	Tyrosine-protein kinase transmembrane receptor Ror2 OS=Drosophila melanogaster GN=Nrk PE=1 SV=2	-0.428565884123491	-0.438307278601691
Q9NJB5	Homeobox protein onecut OS=Drosophila melanogaster GN=onecut PE=2 SV=2	-0.543719518489275	-0.481968507397831
Q9Y171	BcDNA.GH02220 OS=Drosophila melanogaster GN=BcDNA.GH02220 PE=1 SV=1	-1	-0.785875194647153
Q9VKN8	Palmitoyltransferase OS=Drosophila melanogaster GN=Dnz1 PE=1 SV=1	-0.41119543298445	-0.438307278601691
Q9VBF0	CG5447, isoform A OS=Drosophila melanogaster GN=CG5447 PE=1 SV=1	-0.547931769776189	-0.514573172829758
Q6NL34	AT03686p OS=Drosophila melanogaster GN=WDR79 PE=1 SV=1	-0.727379545337008	-0.63486740654747
