# Supplementary material for: An evolutionarily conserved role for separase in the regulation of nuclear lamins
Source: Cell Death Discov. 2025 Oct 21;11:475. doi: 10.1038/s41420-025-02758-5 (PMC12540686; doi:10.1038/s41420-025-02758-5)
Supplement: Supplementary file 5 — Supplementary Table 3 [file 41420_2025_2758_MOESM5_ESM.pdf]

Supplementary Table3. List of differentially expressed proteins in *Sse* mutants after filtering for cell cycle regulators that were also differentially expressed in *dind* mutant. See Text for further details.

Accession	Description	Abundance.Ratio...Sse...Cntrl1	Abundance.Ratio...Sse...Cntrl2
M9NGG5	Futsch, isoform F OS=Drosophila melanogaster GN=futsch PE=1 SV=1	0.683471892855228	0.684369929013007
M9NEP1	Myosin heavy chain, isoform T OS=Drosophila melanogaster GN=Mhc PE=1 SV=1	1,67581593117227	1,67987414774662
M9ND95	Myosin heavy chain, isoform U OS=Drosophila melanogaster GN=Mhc PE=1 SV=1	1,84358192315399	1,77610398807316
E1JHJ3	Myosin heavy chain, isoform O OS=Drosophila melanogaster GN=Mhc PE=1 SV=1	0.624802765252947	0.520045024039818
P05661	Myosin heavy chain, muscle OS=Drosophila melanogaster GN=Mhc PE=1 SV=4	2,68054940871888	2,54695617814141
B7Z001	CG3523, isoform C OS=Drosophila melanogaster GN=FASN1 PE=1 SV=2	0.455228571117139	0.445726703349984
Q7KN85	ATP-citrate synthase OS=Drosophila melanogaster GN=ATPCL PE=1 SV=1	0.438292851579147	0.408168370708106
E2QCF1	ATP-citrate synthase OS=Drosophila melanogaster GN=ATPCL PE=1 SV=1	0.584962500721156	0.491596594410448
P15007	Enolase OS=Drosophila melanogaster GN=Eno PE=1 SV=2	0.480265122054463	0.528071164578735
A0A0B4K7K9	Bruchpilot, isoform J OS=Drosophila melanogaster GN=brp PE=1 SV=1	0.800744623936778	0.838346736521231
D1YSG0	Bent, isoform F OS=Drosophila melanogaster GN=bt PE=1 SV=1	1,16156525639703	1,12035194036522
E1JJA4	Shibire, isoform L OS=Drosophila melanogaster GN=shi PE=1 SV=1	0.836732080459136	0.583037623796664
A0A0B4K843	Bruchpilot, isoform I OS=Drosophila melanogaster GN=brp PE=1 SV=1	0.876566058751721	0.973794929652606
M9PD18	Vacuolar H[+] ATPase 68kD subunit 1, isoform B OS=Drosophila melanogaster GN=Vha68-1 PE=1 SV=1	0.645240512645265	0.608809242675524
A0A0B4KF38	Bruchpilot, isoform M OS=Drosophila melanogaster GN=brp PE=1 SV=1	0.516015147003665	0.699551632523089
X2JDA5	Glutamine synthetase OS=Drosophila melanogaster GN=Gs2 PE=1 SV=1	0.558757430373762	0.54794331129035
M9PEA0	Sallimus, isoform P OS=Drosophila melanogaster GN=sls PE=4 SV=1	0.874993638932967	0.84639302085236
Q9VPV8	IA-2 ortholog, isoform C OS=Drosophila melanogaster GN=IA-2 PE=1 SV=4	0.569491091958716	0.636914580355878
Q8MMD2	Epidermal growth factor receptor pathway substrate clone 15, isoform B OS=Drosophila melanogaster GN=Eps-15 PE=1 SV=1	0.619178216059069	0.58880456701555
P29613	Triosephosphate isomerase OS=Drosophila melanogaster GN=Tpi PE=1 SV=3	0.492622328574446	0.551885103471725
P06754	Tropomyosin-1, isoforms 9A/A/B OS=Drosophila melanogaster GN=Tm1 PE=2 SV=2	1,00216242115062	0.999278472082541
Q7KTI5	CG8086, isoform L OS=Drosophila melanogaster GN=CG8086 PE=1 SV=3	0.696884090855454	0.801572569463598
Q9VNX4	Delta-1-Pyrroline-5-carboxylate dehydrogenase 1, isoform A OS=Drosophila melanogaster GN=P5CDh1 PE=1 SV=1	0.807354922057604	0.784922701562861
Q9VL70	CG4600-PA OS=Drosophila melanogaster GN=yip2 PE=1 SV=1	0.545968369105292	0.553851968181126
M9PEL1	Ras opposite, isoform B OS=Drosophila melanogaster GN=Rop PE=4 SV=1	0.51500591643373	0.566571640626761
Q9VEB1	IP09655p OS=Drosophila melanogaster GN=Mdh2 PE=1 SV=1	0.865522959139964	0.828631581688019
M9PBJ1	Zormin, isoform J OS=Drosophila melanogaster GN=zormin PE=1 SV=1	0.606915941825205	0.654435540845399
E2QCY9	Synapsin, isoform D OS=Drosophila melanogaster GN=Syn PE=1 SV=1	0.687956494044482	0.675364312749146
A0A0B4KHJ9	Tropomyosin 2, isoform E OS=Drosophila melanogaster GN=Tm2 PE=1 SV=1	1,73855168652023	1,79991620298901
Q9VAN7	GH13304p OS=Drosophila melanogaster GN=Pglym78 PE=1 SV=2	0.58880456701555	0.666302128173095
O97477	Inositol-3-phosphate synthase OS=Drosophila melanogaster GN=Inos PE=1 SV=1	0.601221085584946	0.492622328574446
L0MLR4	Calcium/calmodulin-dependent protein kinase II, isoform L OS=Drosophila melanogaster GN=CaMKII PE=1 SV=1	0.696884090855454	0.603121869839996
Q9VBP6	GH21316p OS=Drosophila melanogaster GN=Ssadh PE=1 SV=1	0.40381306161659	0.441483479582645
Q9W401	Probable citrate synthase, mitochondrial OS=Drosophila melanogaster GN=kdn PE=2 SV=1	0.613531652917927	0.577247535593151
Q9VSW2	RE14081p OS=Drosophila melanogaster GN=UGP PE=1 SV=2	0.488515008957812	0.453122446595813
Q9V3N7	BcDNA.HL02693 OS=Drosophila melanogaster GN=CRMP PE=1 SV=1	0.534061602421118	0.499782120147312
Q9W425	Rabconnectin-3A OS=Drosophila melanogaster GN=Rbcn-3A PE=1 SV=3	0.444667066841908	0.404903122145131
B7YZI0	Vacuolar H[+] ATPase 44kD subunit, isoform F OS=Drosophila melanogaster GN=Vha44 PE=1 SV=1	0.540027269257507	0.526068811667588
Q23983	Alpha-soluble NSF attachment protein OS=Drosophila melanogaster GN=alphaSnap PE=1 SV=1	0.605968358841458	0.645240512645265
Q86PA0	CG17816, isoform D OS=Drosophila melanogaster GN=CG17816 PE=1 SV=1	0.464668267003444	0.418999465431266
Q9TVP3	J domain-containing protein OS=Drosophila melanogaster GN=jdp PE=2 SV=2	0.64984535230601	0.692427198089708
A0A0B4K620	Mustard, isoform V OS=Drosophila melanogaster GN=mtd PE=1 SV=1	0.610700062134764	0.564622052436981
P18432	Myosin regulatory light chain 2 OS=Drosophila melanogaster GN=Mlc2 PE=1 SV=2	1,36793014143188	1,44731470025318
A0A0B4KFE4	Acyl-CoA synthetase long-chain, isoform J OS=Drosophila melanogaster GN=Acsl PE=1 SV=1	0.407080775450501	0.423309237242387
E1JIH3	Unc-115a, isoform B OS=Drosophila melanogaster GN=Unc-115a PE=1 SV=1	0.460480470040011	0.435095151620097
P07668	Choline O-acetyltransferase OS=Drosophila melanogaster GN=Cha PE=1 SV=3	0.442545456105304	0.592636428606577
P81900	cAMP-dependent protein kinase type II regulatory subunit OS=Drosophila melanogaster GN=Pka-R2 PE=1 SV=2	0.762986564880576	0.759581973233555
P25455	1-phosphatidylinositol 4,5-bisphosphate phosphodiesterase classes I and II OS=Drosophila melanogaster GN=Plc21C PE=2 SV=3	0.42867840994823	0.435095151620097
Q9VF53	CG18522 OS=Drosophila melanogaster GN=AOX1 PE=1 SV=1	0.407080775450501	0.415758666522498
X2J6D4	Cytochrome c proximal, isoform B OS=Drosophila melanogaster GN=Cyt-c-p PE=3 SV=1	0.614474282837701	0.593592805864596
P54611	V-type proton ATPase subunit E OS=Drosophila melanogaster GN=Vha26 PE=2 SV=1	0.434027674663597	0.404903122145131
E1JJ78	Like-AP180, isoform D OS=Drosophila melanogaster GN=lap PE=1 SV=1	0.742437445376266	0.680774425492461
B5RIU6	AT06279p OS=Drosophila melanogaster GN=EndoA PE=1 SV=1	0.617298482840846	0.682573297347578
M9NE66	Nervous wreck, isoform B OS=Drosophila melanogaster GN=nwk PE=1 SV=1	1,59979385212224	1,33571191032046
Q9VSU8	Nervous wreck, isoform D OS=Drosophila melanogaster GN=nwk PE=1 SV=3	0.715454127115718	0.655351828612554
X2JDD7	Tiggrin, isoform B OS=Drosophila melanogaster GN=Tig PE=1 SV=1	0.867105729502655	0.929033478645856
M9PHR2	Upheld, isoform O OS=Drosophila melanogaster GN=up PE=1 SV=1	1,72071624252113	1,68167414179283
Q9VCF2	Diacylglycerol kinase OS=Drosophila melanogaster GN=CG31140 PE=1 SV=2	0.439357178474257	0.420078115979374
Q95SI7	CG6028 OS=Drosophila melanogaster GN=CG6028 PE=1 SV=1	0.557777671394926	0.525066592078211
Q8SYD9	Endophilin B, isoform A OS=Drosophila melanogaster GN=EndoB PE=1 SV=1	0.718087583960517	0.835924074254375
Q9VNH7	CG2082, isoform A OS=Drosophila melanogaster GN=CG2082 PE=1 SV=1	0.704871964456353	0.727702672837238
A4V4W0	RH38069p1 OS=Drosophila melanogaster GN=stnA PE=1 SV=1	0.697773819555186	0.708407983483596
Q9W058	Succinyl-CoA:3-ketoacid-coenzyme A transferase OS=Drosophila melanogaster GN=SCOT PE=1 SV=1	0.480265122054463	0.495695162624069
Q9VLS7	CG8552, isoform A OS=Drosophila melanogaster GN=PAPLA1 PE=1 SV=1	0.528071164578735	0.462575888042204
Q7KTW5	CG9391, isoform C OS=Drosophila melanogaster GN=CG9391 PE=1 SV=1	0.448900951145128	0.468843942974637
A8JQX3	Curled, isoform D OS=Drosophila melanogaster GN=cu PE=4 SV=1	1,29278174922785	1,28392177230762
Q7K5K3	CG11876, isoform A OS=Drosophila melanogaster GN=CG11876 PE=1 SV=1	0.59741198755465	0.587845009254277
Q9VBA0	CG6330, isoform A OS=Drosophila melanogaster GN=CG6330 PE=1 SV=2	0.67897330785417	0.658097205351372
Q59E09	Acetyl-coenzyme A synthetase OS=Drosophila melanogaster GN=AcCoAS PE=1 SV=2	0.715454127115718	0.590721770009841
Q9VWV5	CG32549, isoform G OS=Drosophila melanogaster GN=CG32549 PE=1 SV=3	0.412510571249805	0.421155960662223
A0A0B4JDC9	RIM-binding protein, isoform F OS=Drosophila melanogaster GN=Rbp PE=1 SV=1	0.446785562143122	0.51500591643373
Q7KVX1	Pyruvate dehydrogenase E1 component subunit alpha OS=Drosophila melanogaster GN=l(1)G0334 PE=1 SV=1	0.522055749160964	0.562669826102702
Q8IPM8	Complexin OS=Drosophila melanogaster GN=cpx PE=2 SV=1	0.574343753920013	0.562669826102702
A8JUZ7	CG15894, isoform B OS=Drosophila melanogaster GN=CG15894 PE=1 SV=1	0.464668267003444	0.586884812852185
Q9V3V2	FK506-binding protein 14 ortholog, isoform A OS=Drosophila melanogaster GN=Fkbp14 PE=1 SV=1	0.509949146304311	0.416839741912829
Q8IM93	CG32017, isoform B OS=Drosophila melanogaster GN=CG32017-RB PE=1 SV=2	0.492622328574446	0.466757615726171
Q9W543	Rabconnectin-3B, isoform A OS=Drosophila melanogaster GN=Rbcn-3B PE=1 SV=1	0.432959407276106	0.454175893185802
P11995	Larval serum protein 1 alpha chain OS=Drosophila melanogaster GN=Lsp1alpha PE=2 SV=3	0.908428650168787	0.900721927564115
O97062	Ccp84Ae OS=Drosophila melanogaster GN=Ccp84Ae PE=1 SV=1	0.656267534794289	0.641546029087524
Q9VM14	AT21758p OS=Drosophila melanogaster GN=CG5261 PE=1 SV=1	0.504874589398464	0.405992359675837
Q95029	Cathepsin L OS=Drosophila melanogaster GN=Cp1 PE=2 SV=2	0.412510571249805	0.459431618637297
P05031	Aromatic-L-amino-acid decarboxylase OS=Drosophila melanogaster GN=Ddc PE=1 SV=4	0.68885174386588	0.708407983483596
Q9VXB0	NECAP-like protein CG9132 OS=Drosophila melanogaster GN=CG9132 PE=2 SV=1	0.492622328574446	0.492622328574446
Q9VZF9	Cuticular protein 64Ad OS=Drosophila melanogaster GN=Cpr64Ad PE=1 SV=2	0.759581973233555	0.833497336859835
Q9VZI3	Unc-112-related protein OS=Drosophila melanogaster GN=Fit1 PE=1 SV=1	0.474046599319306	0.486456955768731
Q9W2M2	Trehalase OS=Drosophila melanogaster GN=Treh PE=1 SV=1	0.819668183496456	0.851998837112446
O96299	LD47736p OS=Drosophila melanogaster GN=Sodh-2 PE=1 SV=1	0.803227036434928	0.679874147746623
Q7K4Q9	HMG coenzyme A synthase, isoform A OS=Drosophila melanogaster GN=Hmgs PE=1 SV=1	0.590721770009841	0.559736524432983
P42281	Acyl-CoA-binding protein homolog OS=Drosophila melanogaster GN=Dbi PE=2 SV=1	0.908428650168787	0.900721927564115
X2JCV2	Larval serum protein 2, isoform B OS=Drosophila melanogaster GN=Lsp2 PE=4 SV=1	0.72159139877538	0.661749599810705
Q9W2J5	CG44245, isoform A OS=Drosophila melanogaster GN=CG44245 PE=1 SV=3	1,09423606984577	1,28214322878150
P82890	Low molecular weight phosphotyrosine protein phosphatase 1 OS=Drosophila melanogaster GN=primo-1 PE=2 SV=1	0.697773819555186	0.722466024471091
A0A0B4LIT4	Microtubule-associated protein OS=Drosophila melanogaster GN=tau PE=1 SV=1	1,10030490579569	1,06557231159362
Q7JRL9	CG31221, isoform A OS=Drosophila melanogaster GN=CG31221 PE=1 SV=1	0.660837367696284	0.644317778337577
Q9VA09	Guanylyl cyclase beta-subunit at 100B OS=Drosophila melanogaster GN=Gycbeta100B PE=1 SV=1	0.528071164578735	0.558757430373762
P14318	Muscle-specific protein 20 OS=Drosophila melanogaster GN=Mp20 PE=2 SV=2	1,13356352574111	1,03068920407114
Q9U6R9	GH13039p OS=Drosophila melanogaster GN=gammaSnap1 PE=1 SV=1	0.560714954474479	0.578214165472454
A0A0B4K7L3	CAP, isoform X OS=Drosophila melanogaster GN=CAP PE=1 SV=1	0.509949146304311	0.50182126542091

A0A0B4KGY6	Pasilla, isoform R OS=Drosophila melanogaster GN=ps PE=1 SV=1	0.583037623796664	0.593592805864596
Q9VWR5	Cytochrome P450 306a1 OS=Drosophila melanogaster GN=phm PE=1 SV=1	0.852797892818771	0.831066510605073
Q9VG55	Protein hugin OS=Drosophila melanogaster GN=Hug PE=1 SV=1	0.571434115876509	0.576280257621355
Q07171	Gelsolin OS=Drosophila melanogaster GN=Gel PE=1 SV=2	1,01077983875324	1,03139519627553
X2JGQ6	CG45057, isoform E OS=Drosophila melanogaster GN=CG45057 PE=4 SV=1	0.773152397014052	0.729444006833663
Q9VIX7	CG15825-PB, isoform B OS=Drosophila melanogaster GN=fon PE=1 SV=1	0.552868871011303	0.540027269257507
Q9W4Y1	CG13759, isoform A OS=Drosophila melanogaster GN=EG:BACR25B3.5 PE=2 SV=2	0.752748591407134	0.848798181244189
O97064	Ccp84Ag OS=Drosophila melanogaster GN=Ccp84Ag PE=1 SV=1	0.461528559472877	0.562669826102702
Q9W0Y1	Troponin C-akin-1 protein OS=Drosophila melanogaster GN=Tina-1 PE=2 SV=1	0.619178216059069	0.558757430373762
Q09024	Neural/ectodermal development factor IMP-L2 OS=Drosophila melanogaster GN=ImpL2 PE=1 SV=4	0.892196710465485	0.978195629681652
Q9VII9	CG31673, isoform A OS=Drosophila melanogaster GN=CG31673 PE=1 SV=2	0.554834395894193	0.471967787661516
A0A0B4LHE7	Vacuolar H[+] ATPase 13kD subunit, isoform B OS=Drosophila melanogaster GN=Vha13 PE=4 SV=1	0.562669826102702	0.633198686374004
Q9VAG9	CG7789 OS=Drosophila melanogaster GN=CG7789 PE=1 SV=1	0.417920007811965	0.421155960662223
Q95NU8	GH16255p OS=Drosophila melanogaster GN=jeb PE=2 SV=1	0.459431618637297	0.506906554580693
Q9W332	Cubilin ortholog OS=Drosophila melanogaster GN=Cubn PE=1 SV=3	0.833497336859835	0.851199338593294
X2JCI6	Troponin C at 73F, isoform C OS=Drosophila melanogaster GN=TpnC73F PE=4 SV=1	1,31498648546852	1,13093086982645
P13217	1-phosphatidylinositol 4,5-bisphosphate phosphodiesterase OS=Drosophila melanogaster GN=norpA PE=1 SV=4	0.497740088609093	0.40381306161659
A8DYP0	Unc-89, isoform C OS=Drosophila melanogaster GN=Unc-89 PE=1 SV=1	0.768925335563751	0.814755482809874
Q9VGH1	Cytochrome P450 315a1, mitochondrial OS=Drosophila melanogaster GN=sad PE=2 SV=1	0.882838655767251	0.869476633965402
Q9VTV9	Delta-aminolevulinic acid dehydratase OS=Drosophila melanogaster GN=Pbgs PE=1 SV=1	0.573374526445944	0.657182660128423
Q7K511	CG3835, isoform A OS=Drosophila melanogaster GN=D2hgdh PE=1 SV=1	0.429749850800216	0.469885976274464
Q9I7J0	CG5023 OS=Drosophila melanogaster GN=CG5023 PE=1 SV=1	1,04893364519792	1,02644598030380
M9PDX2	CG4577, isoform B OS=Drosophila melanogaster GN=CG4577 PE=4 SV=1	0.447843644362086	0.414676780426886
Q9VFC7	Mf5 protein OS=Drosophila melanogaster GN=Mf PE=1 SV=2	0.898401859992193	0.981121989794311
P22979	Heat shock protein 67B3 OS=Drosophila melanogaster GN=Hsp67Bc PE=2 SV=2	0.426533138116673	0.485426827170242
Q9W3M8	CG1515-PA OS=Drosophila melanogaster GN=Ykt6 PE=1 SV=1	0.485426827170242	0.454175893185802
Q9VH98	Diuretic hormone 44, isoform A OS=Drosophila melanogaster GN=Dh44 PE=2 SV=4	0.973060172804084	1,03351110236132
M9PJQ5	Wings up A, isoform K OS=Drosophila melanogaster GN=wupA PE=1 SV=1	1,59645813955899	1,55777767139493
Q9VWD0	GH23568p OS=Drosophila melanogaster GN=parvin PE=1 SV=2	0.51500591643373	0.478195257939166
Q7JYX0	Glutathione S-transferase E14 OS=Drosophila melanogaster GN=GstE14 PE=1 SV=1	0.829443681366591	0.776525151421912
Q9VHC3	Blistery, isoform A OS=Drosophila melanogaster GN=by PE=2 SV=1	0.453122446595813	0.558757430373762
Q9VGF3	CG18547 OS=Drosophila melanogaster GN=CG18547 PE=1 SV=1	0.542010355536609	0.489542935642474
Q9VED8	Deoxyribonuclease II OS=Drosophila melanogaster GN=DNaseII PE=1 SV=1	0.481298941547565	0.43722773891291
Q9VIB5	Carboxylic ester hydrolase OS=Drosophila melanogaster GN=alpha-Est7 PE=1 SV=1	0.916858764699754	0.899948986189672
Q26377	Pro-corazonin OS=Drosophila melanogaster GN=Crz PE=1 SV=2	0.658097205351372	0.693319678811575
Q9VTR6	Pericardin OS=Drosophila melanogaster GN=prc PE=1 SV=2	1,40762467556661	1,38570712465793
Q9W247	CG4752 OS=Drosophila melanogaster GN=CG4752-RA PE=1 SV=2	0.411426245726465	0.526068811667588
Q9V4C1	CG1674, isoform E OS=Drosophila melanogaster GN=CG1674 PE=1 SV=2	1,17184731357334	1,15380533607904
Q9VM18	Trehalose 6-phosphate phosphatase OS=Drosophila melanogaster GN=CG5177 PE=1 SV=1	1,53455968460832	1,59263642860658
Q95TZ7	GH19182p OS=Drosophila melanogaster GN=Zasp66 PE=1 SV=1	1,28806320032532	1,19219416528334
Q9W1D9	Oxysterol-binding protein OS=Drosophila melanogaster GN=CG3860 PE=1 SV=1	0.534061602421118	0.464668267003444
Q8IQX3	CG32544, isoform B OS=Drosophila melanogaster GN=CG32544 PE=1 SV=1	0.496717987935177	0.640620928035698
Q8SZA8	CG1319 OS=Drosophila melanogaster GN=Fdx2 PE=1 SV=1	0.859174455866435	0.972325041557152
P06742	Myosin light chain alkali OS=Drosophila melanogaster GN=Mlc1 PE=1 SV=4	1,45364926604329	1,36569253719753
Q9VVV3	CG14075 OS=Drosophila melanogaster GN=CG14075 PE=4 SV=1	0.620117165028684	0.742437445376266
A0A0B4KGT7	Myosuppressin, isoform B OS=Drosophila melanogaster GN=Ms PE=4 SV=1	0.861558419572029	0.859174455866435
Q9W3L4	CG2233 OS=Drosophila melanogaster GN=CG2233 PE=1 SV=1	2,16059754588055	2,21038886444540
Q9VSN2	CG6416-PA, isoform A OS=Drosophila melanogaster GN=Zasp66 PE=1 SV=1	1,22589186169034	1,39725534559443
Q9W2V2	CG32683, isoform A OS=Drosophila melanogaster GN=CG32683-RA PE=1 SV=2	0.439357178474257	0.504874589398464
M9PDQ9	CG31974, isoform D OS=Drosophila melanogaster GN=CG31974 PE=1 SV=1	0.470927257475127	0.431890348286181
Q9W4W5	CG2680 OS=Drosophila melanogaster GN=EG:100G10.4 PE=1 SV=2	0.679874147746623	0.741574847418796
Q9W3J1	Gbeta5 OS=Drosophila melanogaster GN=Gbeta5 PE=1 SV=1	0.5489297694764	0.506906554580693
Q9VFP6	Inositol polyphosphate 1-phosphatase OS=Drosophila melanogaster GN=Ipp PE=1 SV=1	0.498764146567185	0.425459304765355
Q9VS89	FI18763p1 OS=Drosophila melanogaster GN=frac PE=2 SV=4	0.654435540845399	0.686164326061359
Q9VYA1	CG12177, isoform A OS=Drosophila melanogaster GN=CG12177 PE=2 SV=1	0.530070742225084	0.452068230223811
Q7K0P0	Juvenile hormone-inducible protein 26 OS=Drosophila melanogaster GN=JhI-26 PE=1 SV=1	0.94335876267781	1,2147465226824
Q9VFI3	CG8066, isoform A OS=Drosophila melanogaster GN=CG8066 PE=1 SV=1	0.683471892855228	0.860764202628828
D1FYT3	Odorant-binding protein 99b OS=Drosophila melanogaster GN=Obp99b PE=1 SV=1	0.885183866320351	0.824564212089827
Q9VTJ4	Putative alpha-L-fucosidase OS=Drosophila melanogaster GN=Fuca PE=2 SV=2	0.754459973625479	0.795766947782392
Q9VIF2	CG9248, isoform A OS=Drosophila melanogaster GN=CG9248 PE=1 SV=1	0.429749850800216	0.482332020747376
P92192	Larval cuticle protein 5 OS=Drosophila melanogaster GN=Lcp65Ab1 PE=1 SV=1	2,85319725477036	3,03579983742326
Q9VWD3	CG12531, isoform A OS=Drosophila melanogaster GN=CG12531 PE=1 SV=2	0.401630466584741	0.410341104614087
P47948	Troponin C, isoform 2 OS=Drosophila melanogaster GN=TpnC47D PE=2 SV=2	2,00072116724365	1,96532254836725
Q9W4C1	CG15784, isoform A OS=Drosophila melanogaster GN=CG15784 PE=1 SV=1	1,11370049916473	1,37795663381011
Q9VJD7	CG6639 OS=Drosophila melanogaster GN=SPH93 PE=1 SV=1	2,32250505751888	2,72900887033786
Q9VCU1	CG4721 OS=Drosophila melanogaster GN=CG4721-RA PE=2 SV=2	0.543000877402426	0.584962500721156
Q24400	Muscle LIM protein Mlp84B OS=Drosophila melanogaster GN=Mlp84B PE=1 SV=1	0.859969548221026	0.806530289259566
Q9U9P7	Cytoplasmic phosphatidylinositol transfer protein 1 OS=Drosophila melanogaster GN=rdgBbeta PE=2 SV=1	0.562669826102702	0.504874589398464
D3DML7	MIP14691p OS=Drosophila melanogaster GN=sky PE=1 SV=1	0.408168370708106	0.404903122145131
Q9V3Y7	CG15293, isoform A OS=Drosophila melanogaster GN=CG15293 PE=1 SV=1	0.975263321678493	1,03139519627553
P82147	Protein lethal(2)essential for life OS=Drosophila melanogaster GN=l(2)efl PE=1 SV=1	0.762136169901112	0.762136169901112
Q9W145	Putative cholesterol transporter OS=Drosophila melanogaster GN=Start1 PE=2 SV=2	1,04404433270602	1,14077865578280
A1ZBK7	Crammer OS=Drosophila melanogaster GN=cer PE=1 SV=1	0.711935356978922	0.781569544815974
Q7KTA1	CG31839-PA OS=Drosophila melanogaster GN=NimB2 PE=2 SV=1	0.687060688339892	0.756169328139299
Q8IQ31	CG1695 OS=Drosophila melanogaster GN=CG1695 PE=4 SV=2	0.578214165472454	0.623866861852698
Q5U191	CG1882, isoform A OS=Drosophila melanogaster GN=CG1882 PE=1 SV=1	0.461528559472877	0.551885103471725
P61855	Adipokinetic hormone OS=Drosophila melanogaster GN=Akh PE=1 SV=1	1,34993137336011	1,35501626421955
Q9VLX6	CG7191, isoform A OS=Drosophila melanogaster GN=CG7191 PE=2 SV=3	0.496717987935177	0.561692721398309
O97061	Ccp84Ad OS=Drosophila melanogaster GN=Ccp84Ad PE=4 SV=1	0.409255146684838	0.492622328574446
Q8SXQ5	CG14407 OS=Drosophila melanogaster GN=CG14407 PE=1 SV=1	0.566571640626761	0.519038609600059
P54398	Fat body protein 2 OS=Drosophila melanogaster GN=Fbp2 PE=2 SV=2	1,51450103627429	1,51954190457844
Q8SWS3	CG13049 OS=Drosophila melanogaster GN=CG13049 PE=1 SV=1	0.648925559453121	0.565597175854225
X2JB24	Neuropeptide-like 2, isoform B OS=Drosophila melanogaster GN=Nplp2 PE=4 SV=1	1	1
P92181	CG6956-PA OS=Drosophila melanogaster GN=Lcp65Ac PE=1 SV=1	2	2
P07189	Larval cuticle protein 4 OS=Drosophila melanogaster GN=Lcp4 PE=1 SV=2	2	2
A0A0B4LGF8	Muscle LIM protein at 60A, isoform F OS=Drosophila melanogaster GN=Mlp60A PE=1 SV=1	1	1
A0A0B4LGZ7	CG45076, isoform H OS=Drosophila melanogaster GN=CG45076 PE=1 SV=1	1	1
Q9W306	CG9691, isoform B OS=Drosophila melanogaster GN=CG9691 PE=1 SV=1	1	1
P10552	FMRFamide-related peptides OS=Drosophila melanogaster GN=FMRFa PE=1 SV=2	0.693319678811575	0.789937868980195
Q9VF15	Globin 1, isoform A OS=Drosophila melanogaster GN=glob1 PE=1 SV=1	0.639695233399582	0.579180148127150
Q9VDL4	CG10877 OS=Drosophila melanogaster GN=CG10877 PE=1 SV=1	0.62760683812965	0.632268215499513
M9PE01	Ecdysone-induced protein 63E, isoform N OS=Drosophila melanogaster GN=Eip63E PE=4 SV=1	0.446785562143122	0.479230561206336
Q9VGA3	CG4115 OS=Drosophila melanogaster GN=CG4115 PE=1 SV=2	0.512985334813676	0.625738061908648
Q7JZV0	Cuticular protein 47Eg OS=Drosophila melanogaster GN=Cpr47Eg PE=1 SV=1	2,32308178950373	2,40735275114004
P36188	Troponin I OS=Drosophila melanogaster GN=wupA PE=2 SV=3	1,33799646351502	1,41088377719558
Q9VVE2	Protein rogdi OS=Drosophila melanogaster GN=rogdi PE=1 SV=2	0.685267406516842	0.698662999885884
Q9VGU7	CG14696 OS=Drosophila melanogaster GN=CG14696 PE=1 SV=1	0.422233000683048	0.483364360713349
Q9W5X1	CG9572, isoform A OS=Drosophila melanogaster GN=CG9572-RA PE=2 SV=1	1,18713429147454	1,06143060049775
Q9VGE7	Beta-galactosidase OS=Drosophila melanogaster GN=Ect3 PE=1 SV=1	0.601221085584946	0.591679416935737
A1ZB68	FI01423p OS=Drosophila melanogaster GN=GstE3 PE=1 SV=1	0.768078435016455	0.722466024471091

A0A0B4KFZ3	Phosphodiesterase 6, isoform C OS=Drosophila melanogaster GN=Pde6 PE=4 SV=1	0.535057594894842	0.641546029087524
Q7JVH0	CG8435 OS=Drosophila melanogaster GN=CG8435 PE=1 SV=1	0.598365205323645	0.523060061795249
A0A0B4KF10	Larval cuticle protein 1, isoform B OS=Drosophila melanogaster GN=Lcp1 PE=4 SV=1	2,40381306161659	2,45285896471381
Q9VTP0	CG42255 OS=Drosophila melanogaster GN=CG42255 PE=1 SV=4	0.663572335417523	0.647084212628954
Q9Y136	CG14526 OS=Drosophila melanogaster GN=CG14526 PE=1 SV=2	0.634128557525041	0.753604536279995
Q9VVZ7	CG18294 OS=Drosophila melanogaster GN=CG18294 PE=1 SV=2	0.632268215499513	0.637842060324105
A0A0B4KEF3	Larval cuticle protein 3, isoform B OS=Drosophila melanogaster GN=Lcp3 PE=4 SV=1	1,92067441104162	1,92561971200294
P14199	Protein ref(2)P OS=Drosophila melanogaster GN=ref(2)P PE=1 SV=2	0.511973981781801	0.621993231666123
Q8ML70	Immune-induced peptides OS=Drosophila melanogaster GN=IM10 PE=1 SV=2	1,42921422983958	1,38349694415537
Q9VEK7	Cellular repressor of E1A-stimulated genes, isoform A OS=Drosophila melanogaster GN=CREG PE=1 SV=1	1,35783347862949	1,43935717847426
A0A0B4K679	Rab3 interacting molecule, isoform V OS=Drosophila melanogaster GN=Rim PE=4 SV=1	0.520045024039818	0.499782120147312
Q9VZG1	Cuticular protein 64Ab OS=Drosophila melanogaster GN=Cpr64Ab PE=4 SV=2	0.58880456701555	0.687956494044482
Q9VM58	CG10399, isoform A OS=Drosophila melanogaster GN=CG10399 PE=1 SV=2	0.445726703349984	0.55581615506164
Q9NGX9	Cytochrome P450 302a1, mitochondrial OS=Drosophila melanogaster GN=dib PE=2 SV=2	0.745882688902259	0.825378603892931
Q7K3E2	CG5080, isoform A OS=Drosophila melanogaster GN=CG5080 PE=1 SV=1	0.654435540845399	0.651683180632109
Q9VT29	CG16717 OS=Drosophila melanogaster GN=CG16717 PE=1 SV=1	0.478195257939166	0.519038609600059
Q9VY05	CG9512, isoform A OS=Drosophila melanogaster GN=CG9512 PE=1 SV=1	0.971589535530062	1,14274017211608
Q9VHK7	CG8369, isoform A OS=Drosophila melanogaster GN=CG8369 PE=1 SV=1	0.830255324167683	0.84639302085236
Q7JWW6	Ady43A OS=Drosophila melanogaster GN=Ady43A PE=2 SV=1	0.725959234509188	0.675364312749146
A1Z9F4	CG33156, isoform E OS=Drosophila melanogaster GN=CG33156 PE=3 SV=1	0.794935662803536	0.776525151421912
Q9VLV9	Proctolin OS=Drosophila melanogaster GN=Proc PE=2 SV=2	0.715454127115718	0.738983954700512
Q9W3W4	COQ7 OS=Drosophila melanogaster GN=COQ7 PE=1 SV=2	0.537047519404657	0.43722773891291
Q9VYD5	Branched-chain-amino-acid aminotransferase OS=Drosophila melanogaster GN=CG1673 PE=1 SV=2	1,18142064028014	1,27262045466299
Q9VI25	Neurochondrin homolog OS=Drosophila melanogaster GN=Neurochondrin PE=2 SV=1	0.421155960662223	0.402722176845605
Q8IQU7	825-Oak OS=Drosophila melanogaster GN=825-Oak PE=4 SV=2	0.631337144127481	0.743299527888257
Q9VIQ0	Short neuropeptide F OS=Drosophila melanogaster GN=sNPF PE=1 SV=4	0.621993231666123	0.593592805864596
Q8IPU1	AT19489p OS=Drosophila melanogaster GN=CG33054-RB PE=1 SV=1	0.82781902461732	0.644317778337577
Q9VHK6	CG9836 OS=Drosophila melanogaster GN=IscU PE=1 SV=1	0.539034702970754	0.492622328574446
Q9VCM6	CG4393, isoform B OS=Drosophila melanogaster GN=CG4393 PE=2 SV=4	0.542010355536609	0.53605290024021
Q9VSF2	Mediator of RNA polymerase II transcription subunit 24 OS=Drosophila melanogaster GN=MED24 PE=1 SV=2	0.451013242943973	0.413594082409175
Q9NIP6	Cardio acceleratory peptide 2b OS=Drosophila melanogaster GN=Capa PE=1 SV=1	0.712815854437372	0.743299527888257
M9PF67	CG34356, isoform F OS=Drosophila melanogaster GN=CG34356 PE=4 SV=1	0.411426245726465	0.425459304765355
A0A0B4KEP5	Mitochondrial ribosomal protein S16, isoform B OS=Drosophila melanogaster GN=mRpS16 PE=4 SV=1	1,26963165094044	1,52456522107720
P48596	GTP cyclohydrolase 1 OS=Drosophila melanogaster GN=Pu PE=2 SV=3	0.439357178474257	0.422233000683048
Q03042	cGMP-dependent protein kinase, isozyme 1 OS=Drosophila melanogaster GN=Pkg21D PE=1 SV=2	0.831066510605073	1,04124298223188
M9PF57	CG43897, isoform M OS=Drosophila melanogaster GN=CG43897 PE=1 SV=1	1,69955163252309	1,52155333054509
A0A0B4K6I5	Spartin, isoform B OS=Drosophila melanogaster GN=spartin PE=4 SV=1	1,35614381022528	0.733788168625182
Q86BM0	CG9515, isoform B OS=Drosophila melanogaster GN=CG9515 PE=1 SV=1	0.468843942974637	0.453122446595813
O46100	CG12773, isoform A OS=Drosophila melanogaster GN=EG:8D8.3 PE=1 SV=1	0.477159211186641	0.535057594894842
Q9VQU4	Lectin-24A OS=Drosophila melanogaster GN=lectin-24A PE=2 SV=1	1,41467678042689	1,42061713897869
Q9VLU6	Succinate dehydrogenase assembly factor 4, mitochondrial OS=Drosophila melanogaster GN=Sirup PE=3 SV=2	0.703986603863431	0.73725410432433
Q9V8Y2	General odorant-binding protein 56a OS=Drosophila melanogaster GN=Obp56a PE=1 SV=1	0.639695233399582	0.65901117119815
Q9V3T9	NADPH:adrenodoxin oxidoreductase, mitochondrial OS=Drosophila melanogaster GN=dare PE=2 SV=1	0.544979883255804	0.490570130446201
Q8MLP9	CG30172, isoform A OS=Drosophila melanogaster GN=CSP1 PE=2 SV=1	0.541019153133559	0.526068811667588
Q9W4J9	AT21585p OS=Drosophila melanogaster GN=CG3568 PE=1 SV=2	0.822118274729345	0.657182660128423
A0A0B4KFM8	Metallothionein A, isoform B OS=Drosophila melanogaster GN=MtnA PE=4 SV=1	0.704871964456353	0.739848102699327
Q01583	Diacylglycerol kinase 1 OS=Drosophila melanogaster GN=Dgk PE=2 SV=5	0.583037623796664	0.848798181244189
Q9V773	Probable cytochrome P450 6a20 OS=Drosophila melanogaster GN=Cyp6a20 PE=2 SV=2	0.521050736900963	0.818032474658094
Q9VLJ6	Angiotensin-converting enzyme-related protein OS=Drosophila melanogaster GN=Acer PE=1 SV=1	0.481298941547565	0.43722773891291
P82384	Larval cuticle protein 9 OS=Drosophila melanogaster GN=Lcp9 PE=1 SV=2	2,42304025336151	2,28095631383106
A0A0B4K7U4	Mbl, isoform H OS=Drosophila melanogaster GN=mbl PE=4 SV=1	0.444667066841908	0.58207422139633
Q9XZ56	4E-binding protein THOR OS=Drosophila melanogaster GN=Thor PE=1 SV=1	1,03491998434916	1,46727948045998
H9XVM3	ADP ribosylation factor-like 4, isoform B OS=Drosophila melanogaster GN=Arl4 PE=4 SV=1	0.462575888042204	0.488515008957812
B7Z107	CG42376, isoform A OS=Drosophila melanogaster GN=cin-RB PE=2 SV=1	0.431890348286181	0.473007567916174
B7Z0B0	Sosondowah, isoform G OS=Drosophila melanogaster GN=sowah PE=1 SV=2	0.505890929729957	0.756169328139299
Q9VSU2	Tequila, isoform G OS=Drosophila melanogaster GN=Tequila PE=1 SV=4	1,09963185001446	1,13619138628714
A1Z7R9	CG42382 OS=Drosophila melanogaster GN=rad201 PE=4 SV=1	0.533064921869638	0.542010355536609
A1Z9M6	Vesicular GABA transporter OS=Drosophila melanogaster GN=VGAT PE=1 SV=1	0.585923976958601	0.634128557525041
P02515	Heat shock protein 22 OS=Drosophila melanogaster GN=Hsp22 PE=1 SV=4	1,39396527566024	1,66266125547509
Q9VA42	Niemann-Pick type C-2g, isoform A OS=Drosophila melanogaster GN=Npc2g PE=1 SV=1	0.67626740826589	0.727702672837238
Q7KUD5	Probable insulin-like peptide 5 OS=Drosophila melanogaster GN=Ilp5 PE=1 SV=2	0.848798181244189	0.715454127115718
Q9VQR0	CG3246 OS=Drosophila melanogaster GN=CG3246-RA PE=1 SV=1	0.492622328574446	0.487486349348536
Q9VSY0	Cuticular protein 67B OS=Drosophila melanogaster GN=Cpr67B PE=1 SV=1	0.735522177296537	0.942608336111663
Q7JR83	Hormone-sensitive lipase ortholog, isoform A OS=Drosophila melanogaster GN=Hsl PE=1 SV=1	0.542010355536609	0.524063675777211
A1ZA86	CG8401 OS=Drosophila melanogaster GN=CG8401 PE=4 SV=2	0.439357178474257	0.531069492725954
A0A0B4KGU5	Eclosion hormone, isoform B OS=Drosophila melanogaster GN=Eh PE=4 SV=1	0.537047519404657	0.529071299829111
Q9I7C6	Cuticular protein 65Ax1 OS=Drosophila melanogaster GN=Cpr65Ax1 PE=1 SV=1	1,13158948432814	1,02998286621571
Q9VBJ3	CG42261 OS=Drosophila melanogaster GN=CG42261 PE=4 SV=3	0.528071164578735	0.506906554580693
Q9W4N6	CG6428 OS=Drosophila melanogaster GN=CG6428 PE=1 SV=1	0.409255146684838	0.426533138116673
Q9VPG2	CG13248 OS=Drosophila melanogaster GN=CG13248-RA PE=2 SV=1	0.460480470040011	0.430820496519772
X2JES6	CG1552, isoform C OS=Drosophila melanogaster GN=CG1552 PE=1 SV=1	0.544979883255804	0.82130203988953
Q8IR95	RH08789p OS=Drosophila melanogaster GN=ssp7 PE=1 SV=1	0.521050736900963	0.618238655595455
M9PHG3	Diacylglycerol kinase OS=Drosophila melanogaster GN=rdgA PE=3 SV=1	0.418999465431266	0.535057594894842
Q9VU77	CG10133 OS=Drosophila melanogaster GN=CG10133 PE=1 SV=1	0.760433874670112	0.747602230274943
Q8IP30	CG4793 OS=Drosophila melanogaster GN=CG4793 PE=3 SV=2	2,175684270731	1,973427598004
Q9W293	CG6613 OS=Drosophila melanogaster GN=CG6613 PE=1 SV=1	0.455228571117139	0.457331625485014
Q7JZW0	Cuticular protein 51A OS=Drosophila melanogaster GN=Cpr51A PE=1 SV=1	1,02076886509457	0.937344392150232
Q9W3K9	CG2254-PA OS=Drosophila melanogaster GN=CG2254 PE=1 SV=1	0.504874589398464	0.602171790753379
Q9VD23	Pyruvate kinase OS=Drosophila melanogaster GN=CG7069 PE=3 SV=2	1,20037879798403	0.867896463992655
